# Enhancing upper-limb neurorehabilitation in chronic stroke survivors using combined action observation and motor imagery therapy

**DOI:** 10.3389/fneur.2023.1097422

**Published:** 2023-03-02

**Authors:** Jack Aaron Binks, Jonathan Reyes Emerson, Matthew William Scott, Christopher Wilson, Paul van Schaik, Daniel Lloyd Eaves

**Affiliations:** ^1^Department of Psychology, School of Social Sciences, Humanities and Law, Teesside University, Middlesbrough, United Kingdom; ^2^School of Health and Life Sciences, Allied Health Professions, Teesside University, Middlesbrough, United Kingdom; ^3^School of Kinesiology, University of British Columbia, Vancouver, BC, Canada; ^4^Biomedical, Nutritional and Sport Sciences, Faculty of Medical Sciences, Newcastle University, Newcastle upon Tyne, United Kingdom

**Keywords:** demonstration, neurorehabilitation, brain injury, stroke, motor learning, mental practice, combined action observation and motor imagery (AO + MI) therapy, upper-limb rehabilitation

## Abstract

**Introduction:**

For people who have had a stroke, recovering upper-limb function is a barrier to independence. When movement is difficult, mental practice can be used to complement physical therapy. In this within-participants study we investigated the effects of combined action observation and motor imagery (AO + MI) therapy on upper-limb recovery in chronic stroke survivors.

**Methods:**

A Graeco-Latin Square design was used to counterbalance four mental practice conditions (AO + MI, AO, MI, Control) across four cup-stacking tasks of increasing complexity. Once a week, for five consecutive weeks, participants (*n* = 10) performed 16 mental practice trials under each condition. Each trial displayed a 1st person perspective of a cup-stacking task performed by an experienced model. For AO, participants watched each video and responded to an occasional color cue. For MI, participants imagined the effort and sensation of performing the action; cued by a series of still-images. For combined AO + MI, participants observed a video of the action while they simultaneously imagined performing the same action in real-time. At three time points (baseline; post-test; two-week retention test) participants physically executed the three mentally practiced cup-stacking tasks, plus a fourth unpractised sequence (Control), as quickly and accurately as possible.

**Results:**

Mean movement execution times were significantly reduced overall in the post-test and the retention test compared to baseline. At retention, movement execution times were significantly shorter for combined AO + MI compared to both MI and the Control. Individual participants reported clinically important changes in quality of life (Stroke Impact Scale) and positive qualitative experiences of AO + MI (social validation).

**Discussion:**

These results indicate that when physical practice is unsuitable, combined AO + MI therapy could offer an effective adjunct for neurorehabilitation in chronic stroke survivors.

## Introduction

Stroke is a leading cause of serious long-term disability (1). An acute stroke will reduce the motor ability of around 80% of stroke survivors (2, 3), with the most prevalent physical disability relating to upper-extremity impairments (4–6). Despite the devastating impact that cerebral vascular accidents can have, cognitive neuroscience research shows the brain can reorganize its neural connections in response to learning or experience (7). In the immediate weeks after a stroke, there is a spontaneous clearance of degenerating debris (8), and the neurons that remain attempt to functionally reorganize within the damaged brain area to support, restore and compensate for any function that has already been compromised or lost (9, 10). The central aim of neurorehabilitation is therefore to implement behavioral manipulations (or internal motor simulation processes, for example, if the individual is incapable of physical movement) that encourage the brain to create and reorganize functionally appropriate and relevant neural connections (11).

It is widely accepted that training toward an intended motor outcome (e.g., reaching for, grasping, and transporting a cup) is crucial for stimulating neural plasticity after brain damage and is therefore essential for recovery (11, 12). *Practice* is the key to motor relearning for a stroke survivor; yet for many stroke survivors, physical practice may not be possible or appropriate for relearning lost or impaired skills, since even simple movements can be significantly impaired after stroke (13). It is well documented that experience-dependent learning is essential to help the damaged brain reorganize itself toward functionally relevant recovery (11, 14, 15). While there is heterogeneity in the rate and extent of recovery from stroke, the efficiency and speed of neural reorganization depends on the sensory experiences that can be provided (3, 16).

A large body of research has identified many useful approaches to neurorehabilitation. Maier et al.'s (17) review identified 15 training principles for neurorehabilitation after stroke, based on motor learning and brain plasticity mechanisms. In their review, two mental practice techniques, action observation (AO) and motor imagery (MI), were recommended as useful rehabilitation tools. These two processes evoke an internal motor simulation that has been shown to induce plastic changes, which promote neural connectivity in the motor system (18, 19) and support motor learning (20–22).

AO therapy is well supported as a means to improve motor function in stroke survivors (23, 24). Substantial evidence has confirmed that systematic observation of an action or human movement can prime execution of the same action (25, 26). During observation, a corresponding internal motor representation of the target movement can augment action recognition, imitation, and observational learning (20, 26, 27). To this end, the mirror neuron system's (MNS) capacity to simulate observed actions can be harnessed as a means to restore upper-limb improvement and rearrange compromised neural circuits to rebuild motor function after stroke (15, 28–30). Research shows significant improvements in upper-limb improvement (31, 32), and significant increases in neurophysiological activity in premotor regions, after AO therapy of daily tasks in stroke (33–36).

A substantial body of research has also investigated the potential for MI to promote the relearning of daily tasks following stroke (37, 38). Similar to AO, MI has been found to evoke neural reorganization in a way that corresponds to the effects of physical practice (12, 39–42), and modulates plasticity from cortical to spinal circuitry levels (43, 44). These positive results were supported in Sharma et al.'s (45) fMRI study, which showed that positive changes in connectivity during MI correspond with improved motor function after stroke. Imagery training requires participants to repeatedly form and maintain a motor simulation over time (46, 47). During MI, the brain re-enacts action simulations by creating efferent and afferent activity in the absence of both an accurate external reference (48) and a physical motor output.

Unlike AO therapy, where there is no initial skill requirement, and where unskilled, passive observation can activate motor regions in the brain (27); MI is likely to be a sub-optimal rehabilitation tool for a stroke survivor who is learning complex actions that are absent in their motor repertoire. The brain, without an opportunity to map the observed action, both accurately and reliably, in real time, onto their own sensorimotor system, is likely to default to its own self-developed strategies, driven by its compensatory neural reorganization. This may explain why the evidence for MI benefits in stroke rehabilitation is at best mixed (49–51), and pure MI interventions for stroke survivors frequently do not result in clinically meaningful improvements in upper-limb impairment (52–54).

The advantageous effects of combining AO with MI into a single instruction (AO + MI) are now well documented in neurotypical populations, when compared with the two methods of AO and MI in isolation from one another (55–57). AO + MI therapy involves observing an action whilst simultaneously imagining the kinaesthetic sensations associated with executing the observed action. As such, AO + MI provides a precise external cue which continuously drives an individual's experience-dependent learning, usually resulting in higher self-reported ease of motor-simulation generation (58). In rehabilitation, the proposed advantages for combined AO + MI are predicated on multimodal brain imaging studies (mainly in healthy volunteers), which consistently show AO + MI can produce super-additive effects, compared to either AO or MI, with increased and more widespread activation of motor-related brain regions [e.g., (59–63)]. This combined approach may also reduce the need to understand and follow complex verbal cues–a difficulty many stroke survivors face with MI training (64, 65).

To date, several behavioral studies have explored AO + MI's potential to enhance instantaneous physical outcomes in comparison to both AO and MI instructions in both healthy adults (55, 66, 67) and children (68, 69). To expand this line of work, AO + MI has been found to significantly improve short-term motor learning in comparison to both AO and MI [e.g., one-day; (70); three-weeks; (71), four-weeks; (72), five-weeks; (73), six-weeks; (74–76)]. Most recently, after three consecutive days of AO + MI training (and in the absence of a physical pre-test), Binks et al. (77) used a cup-stacking task in a within-participant design, and showed that AO + MI training significantly reduced movement execution times compared to AO, MI, and an unpractised control condition at both a surprise physical post-test and a one-week retention test. In the present study, we adapted this research design to investigate AO + MI training effects in stroke neurorehabilitation. We also incorporated the same cup-stacking task as in Binks et al. (77), which has been used in previous research to demonstrate improvements in neurorehabilitation via AO therapy in stroke survivors [see (78–80)].

Sun et al. (81) was the first to assess AO + MI training effects in a stroke survivor population. Their study included right-handed participants with right-sided paresis caused by a left hemispheric lesion. Participants imagined grasping, lifting, and inserting a small peg in a hole, before pinching and removing the peg. Half the participants engaged in AO before MI (i.e., asynchronous AO + MI) and the other half performed synchronous AO + MI (i.e., observing an action on-screen, whilst concurrently imagining performing the same action). Training was completed five times per week for four weeks, alongside daily conventional physical rehabilitation. Compared to asynchronous AO and MI (*n* = 5), the synchronous AO + MI therapy (*n* = 5) significantly improved upper-extremity motor function, measured by the Fugl-Meyer Assessment (FMA) and pinch grip strength, while improvements in cortico-motor activation (i.e., electrophysiological activity with greater amplitudes, longer durations, and more frequency components) were also detected for synchronous AO + MI. In a larger study, Choi et al. (82) showed improvements in FMA scores for AO + MI (*n* = 22) compared to AO therapy (*n* = 23) over a five-week period. Those authors further used transcranial magnetic stimulation (TMS) to demonstrate significant changes in corticospinal excitability between pre- and post-tests for the AO + MI but not the AO therapy group. Finally, Robinson-Bert and Woods (83) found significant improvements and minimally important clinical differences in upper extremity motor recovery (FMA scores) for AO + MI practice in sub-acute stroke patients, which incorporated a mean of 5.2 sessions per week for an average period of 2 weeks. This effect only occurred, however, in a sub-group of participants who showed increased commitment to the AO + MI intervention. To extend the approach taken in these three previous studies of AO + MI therapy in stroke survivors (81–83), in the present study we additionally examined motor performance at a two-week retention test.

The overarching research question in the present within-participant study was: can motor simulation enhance motor learning of a novel cup-stacking action in a stroke survivor population? Specifically, our main aim was to quantify the effects of different mental practice conditions (AO + MI vs. AO vs. MI vs. Control) on movement execution times at three time points (baseline vs. post-test vs. retention). The evidence reviewed above indicates clear advantages for AO + MI practice effects both in healthy adults and in stroke survivors, in both the behavioral and neurophysiological measures [see (56, 62)]. We therefore hypothesized in the current study that the combined AO + MI practice condition would reduce movement execution times in the cup-stacking task to a greater extent than in both the AO and MI conditions, and an unpractised control condition, at both the post-test and the retention test. Our secondary aim was to investigate the longitudinal effects of these three mental practice conditions on several additional outcome measures. Liu et al. (84) found that combining AO + MI practice with cognitive training can significantly reduce the effects of vascular cognitive impairments in stroke survivors, compared to when using cognitive training alone, as indicated by the Montreal Cognitive Assessment Scale. On these grounds, we explored whether the AO + MI practice administered in the current study would also improve health-related quality of life, MI ability and upper limb performance. We also monitored self-reported imagery use over time (i.e., to check compliance with the intervention) and we investigated the participants' qualitative experiences of the experimental conditions.

## Materials and methods

### Participants

Participants were recruited from a community-led stroke group in the North East of England (*n* = 10, *M* age = 64.4 years, *SD* = 9.4, males = 6, see Table 1). All participants were volunteers and informed of the screening protocol before participation. Inclusion criteria for participation included: (1) clinical diagnosis of stroke of any etiology; (2) a minimum of 6 months post-stroke onset; (3) <75 years old; (4) normal or corrected-to-normal vision (i.e., no hemianopsia); (5) no prior experience of a MI intervention.

**Table 1:** Stroke demographic information.

**Participant**	**Sex**	**Age**	**Lesion**	**Side**	**Days between first stroke onset and participation**	**Hands used in task**
1	M	72	RT intracranial hemorrhage frontal lobe	LT	745	1
2	M	75	LT lacunar infarct	RT	881	2
3	F	58	RT hemorrhage lentiform nucleus	LT	1,114	1
4	M	64	LT small focus of restricted diffusion to medullary pyramid	RT	1,631	2
5	F	43	RT cerebral peduncle infarct	LT	1,575	2
6	M	61	RT thalamic ischaemia	LT	1,755	1
7	F	66	LT total anterior circulation infarction	RT	1,310	2
8^*^	F	73	RT basal ganglia subinsular infarct with hemorrhagic transformation	LT	1,676	1
9^*^	M	70	LT total anterior circulation infarction	RT	668	2
10^*^	M	62	RT thalamic ischaemia	LT	2,098	1
M (SD)		64.40 (9.40)			1,345.30 (479.30)	

The sex, age, lesion location, lesion hemisphere, number of days from first stroke onset to participation in the study, and number of hands used in the experimental cup-stack task. For each participant who completed the study.

^*^Denotes three participants who did not complete the training phase and did not report post and retention test scores.

The exclusion criteria included the following: (1) moderate pain in the affected limb (> 5 on the Visual Analog Numeric Pain Distress Scale; VAS 1–10 pain scale); (2) complete paralysis of the affected limb (any participant who could not voluntarily generate a minimum of 10° flexion at the radiocarpal, metacarpophalangeal and interphalangeal joints); (3) severe cognitive dysfunction (<8/10 on Kingshill Version 2000 of the 6CIT; 85); (4) hemineglect; (5) moderate or severe aphasia; or (6) reduced MI ability [<5/7 in the non-paretic limb and <4/7 in the paretic limb, using a modified version of the Motor Imagery Questionnaire-3; MIQ-3; (86)]. Responses for the MIQ-3 at the baseline confirmed a good capacity for MI in the non-paretic limb (*M* = 5.54, *SD* = 0.88) and the paretic limb (*M* = 4.45, *SD* = 1.60), and across the following imagery types: internal (1st person) visual perspective (*M* = 5.29, *SD* = 1.19), external (3rd person) visual perspective (*M* = 4.96, *SD* = 1.42), kinaesthetic imagery (*M* = 4.74, *SD* = 1.57). To further evaluate the clinical status of participants, the Action Research Arm Test [ARAT; (87)] and the Stroke Impact Scale [SIS; (88)] were also completed at the baseline.

Screening of eleven participants, using the above criteria, permitted 10 participants for inclusion (see Table 1). These ten participants were included in the analysis of the baseline data, while three were removed from the analyses of the post-test and retention test data due to drop-out. All participants provided written informed consent in accordance with ethical clearance from the local research ethics committee.

### Task and research design

**Figure 1:**
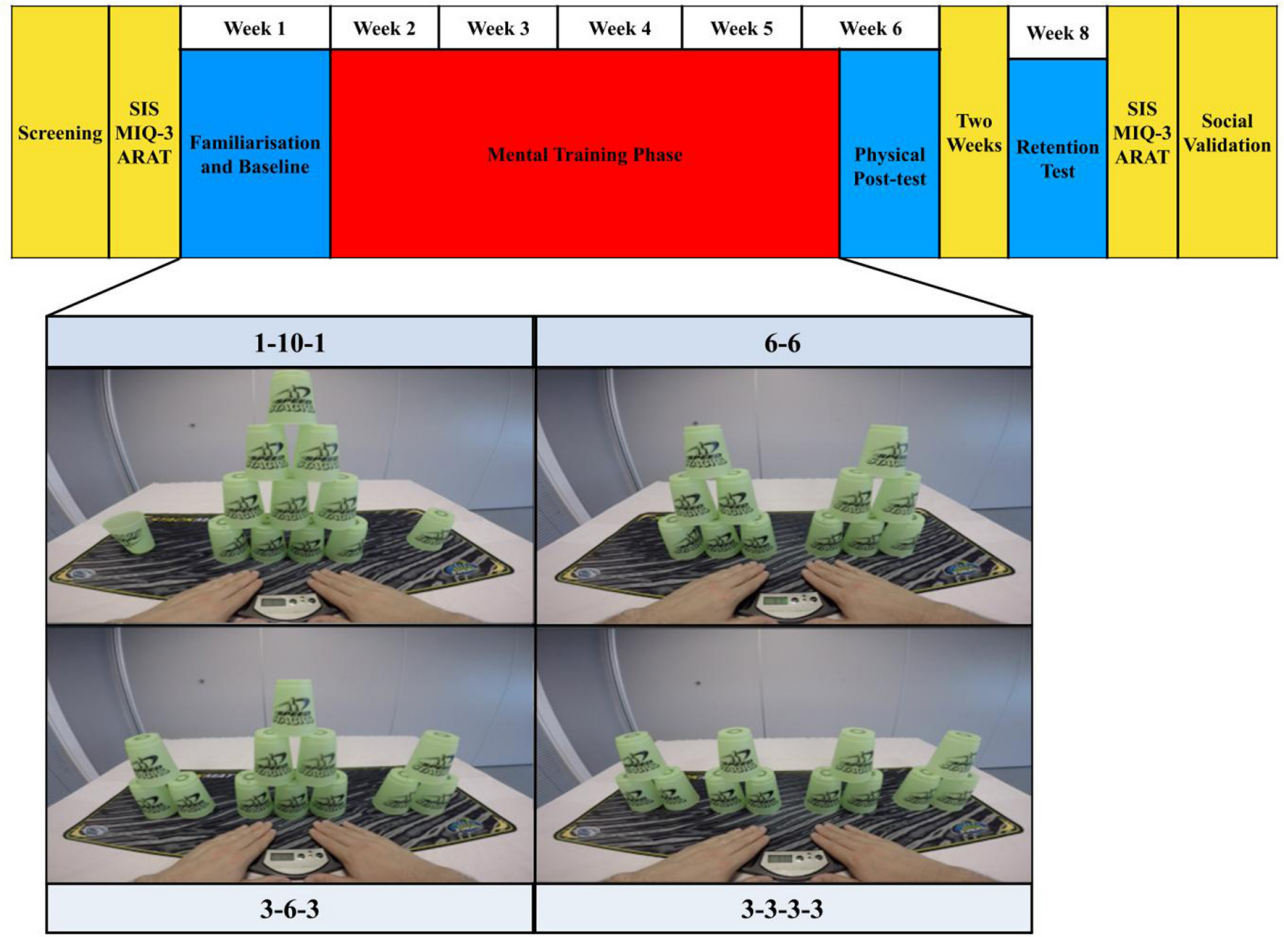
Research design and task. The structure of the research design is displayed across the top of this figure. On each practice day, the four cup-stack sequences shown (tasks) were paired with the following practice conditions in a fully counterbalanced order across participants: action observation (AO), motor imagery (MI), motor imagery during action observation (AO + MI) and an unpractised control. On each practice day, participants experienced 16 trials, lasting 5 min in total, per practice condition. They physically executed five repetitions of each task at the baseline, post-test, and two-week retention test. Key for additional measures: SIS, Stroke impact scale; MIQ-3, Motor Imagery Questionnaire 3; ARAT, Action Research Arm Test.

After undertaking the screening and a familiarization session, participants completed a baseline test involving physical execution of four different cup-stacking sequences, as fast and as accurately as possible (see Figure 1). The main dependent variable was the time taken to complete the physical execution of each cup-stacking task. These data were recorded at the baseline (Week 1), post-test (Week 6) and in a retention test (Week 8). All participants were instructed to maintain their normal daily activity routines throughout the duration of the experiment. Moreover, participants were instructed to not physically practice the cup-stacking tasks outside of the current experiment.

In the training phase (Weeks 2–6), participants experienced three practice conditions: action observation (AO), motor imagery (MI) and combined action observation and motor imagery (AO + MI). While the unpractised control was omitted during the training phase, participants watched (AO), imagined (MI) and simultaneously watched and imagined (AO + MI) three, randomly assigned, counterbalanced cup-stacking sequences (task) once a week for five consecutive weeks. The task used as the unpractised control sequence was physically executed in Week 1, 6, and 8.

As in the study by Binks et al. (77), a within-subjects, repeated-measures, Graeco-Latin square design was used to randomly assign a pairing between each level of the Graeco factor of “task” (involving four levels of cup-stack sequence: 1-10-1, 6-6, 3-6-3, 3-3-3-3) and the Latin factor of “practice condition” (AO, MI, AO + MI, Control). In addition, the Graeco-Latin square allowed investigation and control of two other *blocking* factors, namely: “presentation order” (Order 1, 2, 3, 4) and “group” (Group 1, 2, 3, 4). This four-factorial design was necessary to counterbalance the four levels of the four factors (i.e., task and practice condition across group and presentation order). A random permutation of this design resulted in 16 unique task and practice condition pairings. Each pairing occurred exactly once in each group and presentation order [see Table 2; (89)]. This is an efficient design approach to study the effect of one treatment factor in the presence of three extraneous variables (90). In the context of motor learning, this is particularly useful as the design completely randomizes the presentation order for each practice condition using a within-participant design. Research has also shown that the Graeco-Latin-square design is typically more efficient and hence more powerful than reasonable alternatives (91, 92).

**Table 2:** Graeco-Latin square design.

	**Order 1**	**Order 2**	**Order 3**	**Order 4**
Group 1	AO + MI *(1 − 10 − 1)*	AO *(6 − 6)*	MI *(3 − 6 − 3)*	Unpracticed *(3 − 3 − 3 − 3)*
Group 2	AO *(3 − 3 − 3 − 3)*	AO + MI *(3 − 6 − 3)*	Unpracticed *(6 − 6)*	MI *(1 − 10 − 1)*
Group 3	MI *(6 − 6)*	Unpracticed *(1 − 10 − 1)*	AO + MI *(3 − 3 − 3 − 3)*	AO *(3 − 6 − 3)*
Group 4	Unpracticed *(3 − 6 − 3)*	MI *(3 − 3 − 3 − 3)*	AO *(1 − 10 − 1)*	AO + MI *(6 − 6)*

This sophisticated tool is designed to systematically control three sources of extraneous variability (the row blocking factor of group; the column blocking factor of presentation order and the Graeco blocking factor of task). This provides the opportunity to investigate the effects of one treatment factor (i.e., practice condition), that is fully counterbalanced across the other blocking factors [see (89)].

On each of the 5 days of the training phase, participants undertook 3 blocks of mental practice trials (AO, MI, and AO + MI; each paired with a different task across participants). Participants received short rests between each block of trials. Blocks consisted of 16 trials lasting 5 min each.

In addition to the movement execution times, the following measures were recorded in the week before the baseline and the week after the retention test: the MIQ-3, the ARAT, and the SIS. On Weeks 2, 4, and 6 in the training phase we also tracked the participants' imagery use. A qualitative interview (social validation) was conducted at the retention test to explore the participants' perceptions of the training phase.

### Stimuli and apparatus

The present study contained four cup-stack sequences. Three of these sequences were approved World Sport Stacking Association (WSSA) stacks: 1-10-1, 6-6 and 3-6-3. The fourth sequence was adapted from the WSSA 3-3-3 to a 3-3-3-3; this adjustment ensured 12 cups were presented in each sequence (see Figure 1).

**Figure 2:**
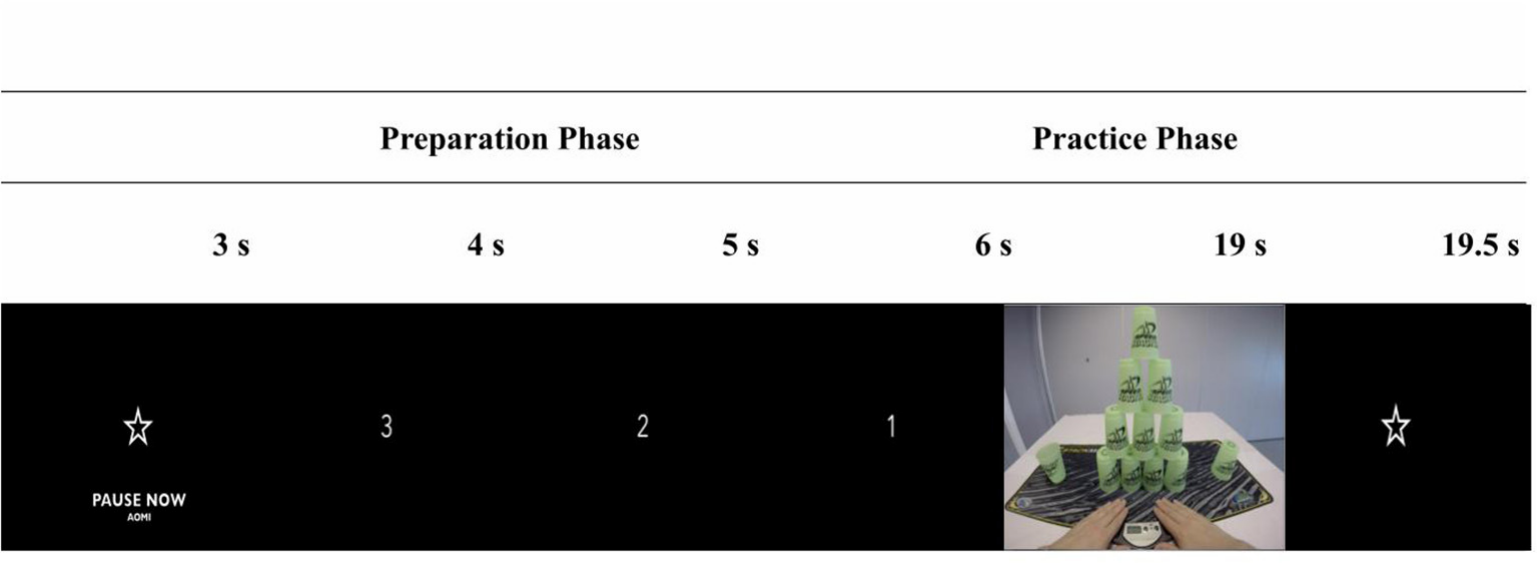
Trial sequence example. This figure shows the structure on each AO + MI practice trial.

A desk-mounted video camera (GoPro Hero 4; GoPro.com, 2016) was used to record each cup-stacking sequence from a 1st person visual perspective. All sequences were recorded in a laboratory setting, with an immersive visual dimension of 1,920 × 1,080 p, shot at 30 frames per second. Each sequence was 13 s in length and the videos were edited in iMovie (Apple, New York, NY). Each trial began with a white star on a black screen (3 s), a ‘3, 2, 1' countdown (3 s), followed by exposure to the cup-stacking sequence (13 s); totalling 48 trials per day (Figure 2).

These videos were initially recorded and used in the study by Binks et al. (77) to display each cup-stacking sequence executed over an 8 s period. In the current study, pilot testing determined that it was necessary to slow these videos down to 60% of the original speed, so that the action in the video (lasting 13 s) would be executed at a pace that was realistic for imitation in this population. Each cup-stacking trial showed two hands lifting from a pressure-sensitive timing pad (Pro-Timer; StackMat^®^™) to reach forward and pick up a vertical column of stacked cups. The task required participants to “up-stack” the cups from left to right in a predetermined sequence. Once the sequence was complete the cups were “down-stacked” from right to left into their original positions. The task was completed when the hands returned to the pad. During each training session participants sat at a desk, in a dimly lit room, facing a 13.3-inch LED-backlit monitor display (Apple, New York, NY).

### Procedure

#### Familiarization and baseline

After screening and 1 week before the familiarization and baseline test, the MIQ-3, the ARAT, and the SIS data were recorded (see Figure 1). The familiarization session required participants to complete all four cup-stacking sequences in order of difficulty (i.e., 3-3-3-3, 3-6-3, 6-6 and 1-10-1). At a desk, sitting opposite the participant, a researcher first provided guided verbal and visual instruction for the completion of each full cup-stack sequence. Feedback was provided to confirm that participants had established the correct technique. The participant successfully completed each task once with assistance and once without. Participants were also instructed how to use the pressure-sensitive timing pad.

Upon completion of the familiarization, participants were randomly assigned into one of four experimental groups. Each group contained a different practice condition and task combination for each participant to mentally practice throughout the training phase (see Table 2). To record a baseline score in Week 1, each participant had three attempts to complete each task as quickly and as accurately as possible. This is a complex and controlled motor sequencing task, wherein any movement errors would be reflected in the time taken to complete the movement [Foerster et al., (93), p. 201)]. If an error was made (e.g., a cup was dropped), participants were asked to correct their error and continue the cup-stacking task until the sequence was complete.

To familiarize participants to the imagery and observation instructions used in the main training phase, each participant was guided through a training video which presented a simplified and slowed cup-stack sequence. Accompanied with verbal guidance, this phase built a foundational understanding of how the imagery and observation instructions were to be integrated into each cup-stacking sequence during the five-week training phase. All participants were instructed to not physically or mentally practice the tasks outside of scheduled sessions.

#### Main experiment and five-week training phase

##### Action observation

**Figure 3:**
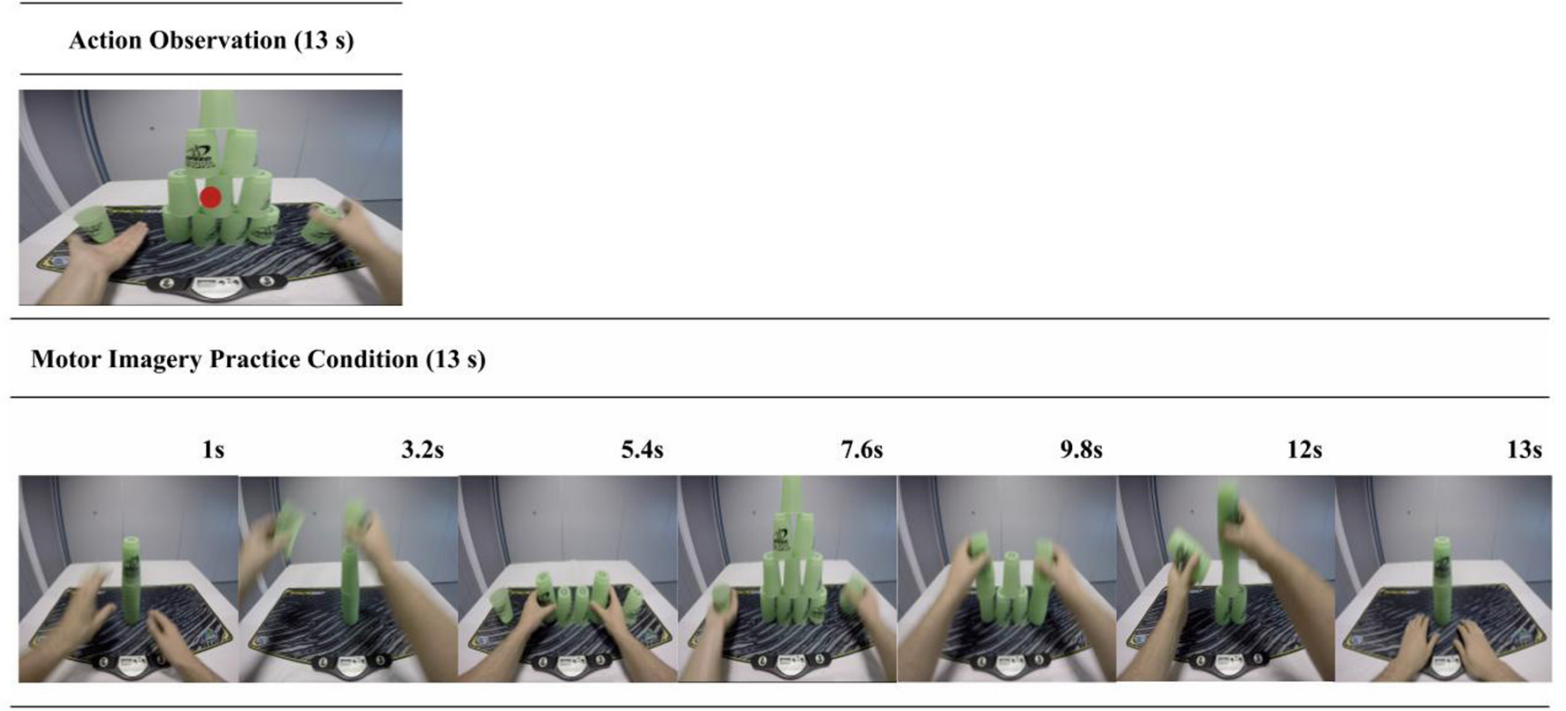
AO and MI trial sequence example. This figure shows the structures for both the AO (**top** panel) and the MI (**bottom** panel) practice trials. In the AO condition participants verbally reported the color of a dot that appeared at the midpoint of the video in 25% of the trials. This aimed to control for attention toward the display and reduce the potential confound of spontaneous or unintended MI during the pure AO condition. In the MI condition participants viewed a series of still-images portraying a cup-stack sequence. These were designed to both cue and control for the time duration of the MI.

Participants were instructed to watch the on-screen cup-stacking sequence while refraining from using any MI. Participants were asked to attend only to the occasional appearance of a colored dot and, when it appeared, to inform the researcher what color it was. Participants were naïve to the fact the dot would only appear on trials 1, 5, 10, and 15 and that the presentation color alternated between red and blue. The dot appeared in the middle of the screen, for 0.5 s, at the point of transition between the up-stack and down-stack, when the observed hands were not touching the cups (see Figure 3). The colored dot was precisely integrated into this practice condition to reduce the potential of spontaneous or unintentional MI [see (57)]. This simple task motivated participants to engage with the videos without distracting them from the observed action.

##### Motor imagery

Participants viewed a series of still-images portraying a cup-stacking sequence (see Figure 3), which visually depicted the stages of completing the sequence. The MI practice condition was administered in this way to provide basic visual cues to help structure and sequence their MI without providing observation of a dynamic action. These instructions also incorporated some of the PETTLEP principles (94–96). During each trial, participants were instructed to imagine performing the action in a 1st person *perspective* and to maintain an emphasis on “feeling” the imagined movement [e.g., (97)]. Participants imagined performing the task within the experiment *environment*, while in a similar *physical* state as would be adopted during performance (i.e., seated at a desk). Participants were guided to imagine the *timing* of the action in accordance with the sequence of on-screen pictures. They were asked to recreate the *task* specific components of reaching, grasping, placing and releasing the cups in a specific order.

##### Action observation during motor imagery

This entailed imagining the sensation and kinaesthetic experience of executing the action and synchronizing this motor simulation with the congruent observed action (55). Similar to the MI instruction, some PETTLEP components were incorporated into the AO + MI delivery (96). As in the MI condition, participants were instructed to specifically focus on imagining the kinaesthetic sensation involved in performing the observed *task* sequence from a 1st person visual *perspective*. While seated at a desk (*physical*), they were additionally instructed to imagine themselves performing this action at the speed presented on screen (*timing*).

##### Unpractised control

Upon completion of the familiarization and baseline test, one cup-stacking sequence, assigned as the unpractised control, was not presented to the participant again until the physical post-test at Week 6. Due to the random assignment of the Graeco-Latin square, a different cup-stack sequence was omitted in each experimental group.

#### Post-test and retention test

The post-test (administered on Week 6, immediately after completion of the training phase) required participants to physically execute each of the cup-stacking sequences in the same order as they had experienced them at baseline and on each practice day throughout the training phase (see Table 2). The fourth unpractised cup-stack sequence (Control) was also reintroduced. The retention test required participants to replicate all procedures administered at the post-test. After the retention test, participants again completed the MIQ-3, ARAT, SIS, and completed a social validation interview. On Weeks 2, 4, and 6 participants also completed an adapted self-reported questionnaire to track imagery use. The approach to these additional measures is described below.

#### Additional outcome measures

##### Perceived impact of stroke

The perceived impact of stroke was assessed using the Stroke Impact Scale [SIS, (98)], a self-report measure recorded at the baseline and retention test. This tool evaluates disability and health-related quality of life after stroke. The sub-categories for assessment are everyday functioning in: strength, memory, emotions, communication, activities of daily living and instrumental activities of daily living (ADL/IADL), mobility, hand function, participation and total stroke recovery. The stroke impact scale has been found to have high levels of internal consistency in the UK (99).

##### Motor imagery ability

To assess MI ability, participants completed the MIQ-3 measure at the baseline and retention test. This measure has good psychometric properties, internal reliability, and predictive validity (86). Participants self-reported the ease with which they could generate imagined actions, such as a cup lift and arm abduction (1 = very hard to see/feel; 7 = very easy to see/feel) on three subscales: internal visual imagery, external visual imagery, and kinaesthetic MI.

##### Upper extremity performance

The Action Research Arm Test (ARAT) was used to assess upper extremity performance (coordination, dexterity, and functioning) in stroke recovery between the baseline and retention test. Originally described by Lyle (87) as a modified version of the Upper Extremity Function Test, this is a 19-item observational measure. These items are categorized into four subscales (grasp, grip, pinch, and gross movement) and arranged in order of decreasing difficulty, with the most difficult task examined first, followed by the least difficult task. Task performance is rated on a 4-point scale, ranging from 0 (no movement) to 3 (movement performed normally). Nijland et al. (100) found the internal consistency of the ARAT using Cronbach's Coefficient Alpha as excellent (α = 0.98), while Inter-rater reliability, as analyzed using the inter correlation coefficient (ICC) was also excellent (ICC = 0.92).

##### Self-reported imagery use over time

A questionnaire was adapted from the established MIQ-3. Our questionnaire was administered after every block of MI and AO + MI trials on each day of practice on Weeks 2, 4, and 6. Participants self-reported their ease of imagery generation on a 1–7 Likert scale (1 = very hard to see/feel or very unconfident, 7 = very easy to see/feel or very confident). The original MIQ-3 item “Kinaesthetic imagery” was retained in the current study to rate the ease of generating the feeling and effort of imagined cup-stacking. The original questionnaire also requires participants to rate visual imagery separately for both an internal (1st person) and external (3rd person) visual perspective. In the current study, we instead used the generic item “ease of generating visual imagery” and then required participants to indicate “perspective used” (internal or external). This assessed the ease or difficulty of generating the visual components of the imagined cup-stacking task and additionally allowed us to monitor changes in their preferences for visual perspective over time.

Finally, the question: “how confident were you that no type of imagery was used?” was asked after each block of AO. This measure was utilized to monitor and assess any potential spontaneous or unintended MI during the pure AO condition. If any participant reported <3 on the 1-7 Likert scale to indicate that they were: (1) “very unconfident” (2) “unconfident” or (3) “somewhat unconfident” that no type of imagery was used, they additionally completed the adapted imagery questionnaire (described above) for the AO condition.

##### Social validation

Immediately after completing the retention test, the primary researcher conducted a semi-structured social validation interview with each participant to check for compliance with the intended manipulations and gauge their experiences of the experimental conditions. The interview guide included 10 initial questions (e.g., “Do you have any comments on the difficulty of performing AO, MI or AO + MI?”). Follow-up probes were listed for each question to gain the necessary detail from all participants (e.g., “What made this task difficult for you?”, “Was this task easier or harder than the other experimental tasks, and why do you think this was the case?”). Questions explored the perceived ease and use of the imagery and observation instructions. The questions also targeted overall effect, attention (direction and level), unintentional or spontaneous imagery and which instruction modality the participant liked more or felt most confident and comfortable using. The interview ended with advice on what future imagery and observation interventions should entail.

##### Attentional errors

When the “3, 2, 1” countdown was shown on the computer screen, participants were instructed to place their hands on the timing pad in front of them. While their forearms rested on the desk, participants were required to lift both hands off the pad in synchrony with the hands presented on the display. The time taken between their hands leaving and returning to the pad was recorded for each trial. This reflected the time spent on each trial imagining, observing, or both imagining and observing a cup-stacking sequence. Each stimulus presentation lasted 13 s; therefore, periods of time <12.5 s or >13.5 s (recorded on the timer) were counted as an attentional error. Across the practice phase each participant completed 80 trials in each practice condition (240 trials in total). All practice conditions contained <5% errors meaning that out of 80 trials all participants recorded a minimum of 76 trials.

#### Data analysis: Movement execution times

The main dependent variable was the time taken to complete the cup-stacking movement. When participants lifted their hands from the pressure pad, the timer ran until they had completed the full sequence of the cup-stack task and returned their hands on the pad.

All analyses were performed in the statistical package R 4.1.0 (101). The R package *lme4* was used for the construction and analysis of the linear-mixed-model of the four-factor Graeco-Latin square design. While the robustness of mixed-effects models is established (102) along with use in small samples (103), distributional assumptions were also considered using the *performance* package. For each stage of the analysis of the movement time data and at each of the three time points (baseline, post-test, and retention test) a mixed-effects model was tested, with participant included as the random factor. The fixed factors were the Graeco factor of task, the Latin factor of practice condition, and the blocking factors were presentation order and group. The design was carried through into the analysis of the results (104). The interactions of time point (baseline, post-test, and retention test), with the fixed factors, were then added and the delta-Akaike information criterion (ΔAIC) was used to evaluate the difference in AIC scores between the two models.

*Post-hoc* results were averaged over the levels of group, order, and task. At *post-hoc* maximum likelihood estimates of the parameters of the linear mixed model, including the method for computing the denominator degrees of freedom and F-statistics, were determined using Satterthwaite's method (105). Type III sums of squares were used in significance-testing. The significance level was set to 0.05 and effect sizes were calculated as partial eta squared values ($\eta_{p}^{2}$); values of 0.0099, 0.0588, and 0.1379 were used as benchmarks for small, medium, and large effect sizes (92) as suggested by Cohen [(106), p. 278–280].

#### Interaction effects within the Graeco-Latin square design

In accordance with conventional approaches to the Graeco-Latin square design and analysis it was not appropriate to explore the interaction effects *within* our main data set (i.e., only interactions involving the factor of time are permitted, since this factor is not included in the Graeco-Latin square design). An assumption of the Graeco-Latin square design is that of a null main effect for the Graeco factor (i.e., cup-stacking sequence) which according to Kohli (107), does not permit a useful interpretation of the related interactions between the treatment factor (practice condition) and each of the other blocking factors (group and presentation order).

#### Data analysis: Additional outcome measures

##### Perceived impact of stroke

Minimal detectable change and clinically important differences were assessed for the baseline vs. retention test in the eight domains and in total stroke recovery.

##### Motor imagery ability

A multi-factorial analysis of variance (ANOVA) was used to analyze the effects of limb (paretic vs. non-paretic), MI sub-scale (kinaesthetic vs. internal 1st person visual vs. external 3rd person visual perspective), and time (baseline vs. post-test vs. retention test).

##### Upper extremity performance

A two-factorial ANOVA was run involving the factors of limb (paretic, non-paretic) and time (baseline, retention test).

##### Self-reported imagery use over time

Descriptive data for MI use are presented as mean and SD scores for each mental practice condition (AO, MI, and AO + MI) at three time points (Weeks 2, 4, and 6), with user preferences for visual perspective (internal, external), and the perceived frequency of spontaneous MI during the AO condition.

##### Social validation

Qualitative interview data were interpreted using Braun and Clarke's (108) six-step thematic analytical procedures. The data analysis involves: (1) familiarization with the data, (2) transcription of the audio recorded interviews, (3) identification of the initial codes, (4) identification of themes, (5) naming, reorganizing, and completing the themes and (6) theme comparison and write-up.

## Results

### Baseline

There was no significant main effect of practice condition on movement execution times at the baseline test, *F*_(3, 110)_ = 1.07, *p* = 0.36, $\eta_{p}^{2}$ = 0.03. There was, however, a significant main effect of task, *F*_(3, 110)_ = 21.72, *p* < 0.001, $\eta_{p}^{2}$ = 0.37. From slowest to fastest: 1-10-1 (*M* = 60.62, *SD* = 44.82) > 6-6 (*M* = 47.12, *SD* = 39.91, *t*(120) = 3.00, *p* = 0.05) > 3-6-3 (*M* = 37.67, *SD* = 24.74) > 3-3-3-3 (*M* = 28.41, *SD* = 25.85). While 6-6 did not significantly differ from 3-6-3, nor 3-6-3 from 3-3-3-3 all other task combinations significantly differed at *p* < 0.001.

There was a significant main effect of presentation order, *F*_(3, 110)_ = 5.98, *p* < 0.001, $\eta_{p}^{2}$ = 0.14. Order 1 (*M* = 51.79, *SD* = 47.49) was significantly slower in comparison to Order 3 (*M* = 37.73, *SD* = 23.57, *t*(120) = 3.01, *p* = 0.05) and Order 4 (*M* = 36.51, *SD* = 25.34, *t*(120) = 2.91, *p* = 0.05). Order 2 (*M* = 47.78, *SD* = 42.91) recorded the second slowest movement execution times and was significantly slower in comparison to Order 3, *t*(120) = 2.75, *p* = 0.05. All other presentation orders were not significantly different from each other. Finally, there was no significant main effect of group, *F*_(3, 110)_ = 1.17, *p* = 0.37, $\eta_{p}^{2}$ = 0.26.

### Post-test

There was no significant main effect of practice condition on movement execution time at the post-test, *F*_(3, 77)_ = 1.77, *p* = 0.16, $\eta_{p}^{2}$ = 0.06. A significant main effect of task was present at the post-test, *F*_(3, 77)_ = 58.34, *p* < 0.001, $\eta_{p}^{2}$ = 0.69. As at the baseline, the cup-stacking sequences differed from each other in the following order: 1-10-1 (*M* = 33.12, *SD* = 10.43) > 6-6 (*M* = 26.57, *SD* = 9.82, *t*(40) = 3.88, *p* < 0.01) > 3-6-3 (*M* = 22.60, *SD* = 7.73, *t*(40) = 3.18, *p* < 0.01) > 3-3-3-3 (*M* = 16.96, *SD* = 5.34, *t*(87.2) = 4.26, *p* < 0.001). All comparisons were significantly different from one another, *p* < 0.001. There was no significant main effect of presentation order, *F*_(3, 77)_ = 1.61, *p* = 0.19, $\eta_{p}^{2}$ = 0.06. There was a significant main effect of group, *F*_(3, 7)_ = 9.69, *p* < 0.01, $\eta_{p}^{2}$ = 0.81. However, upon completion of *post hoc* analysis no specific pairwise comparisons were significantly different from one another.

### Retention test

**Figure 4:**
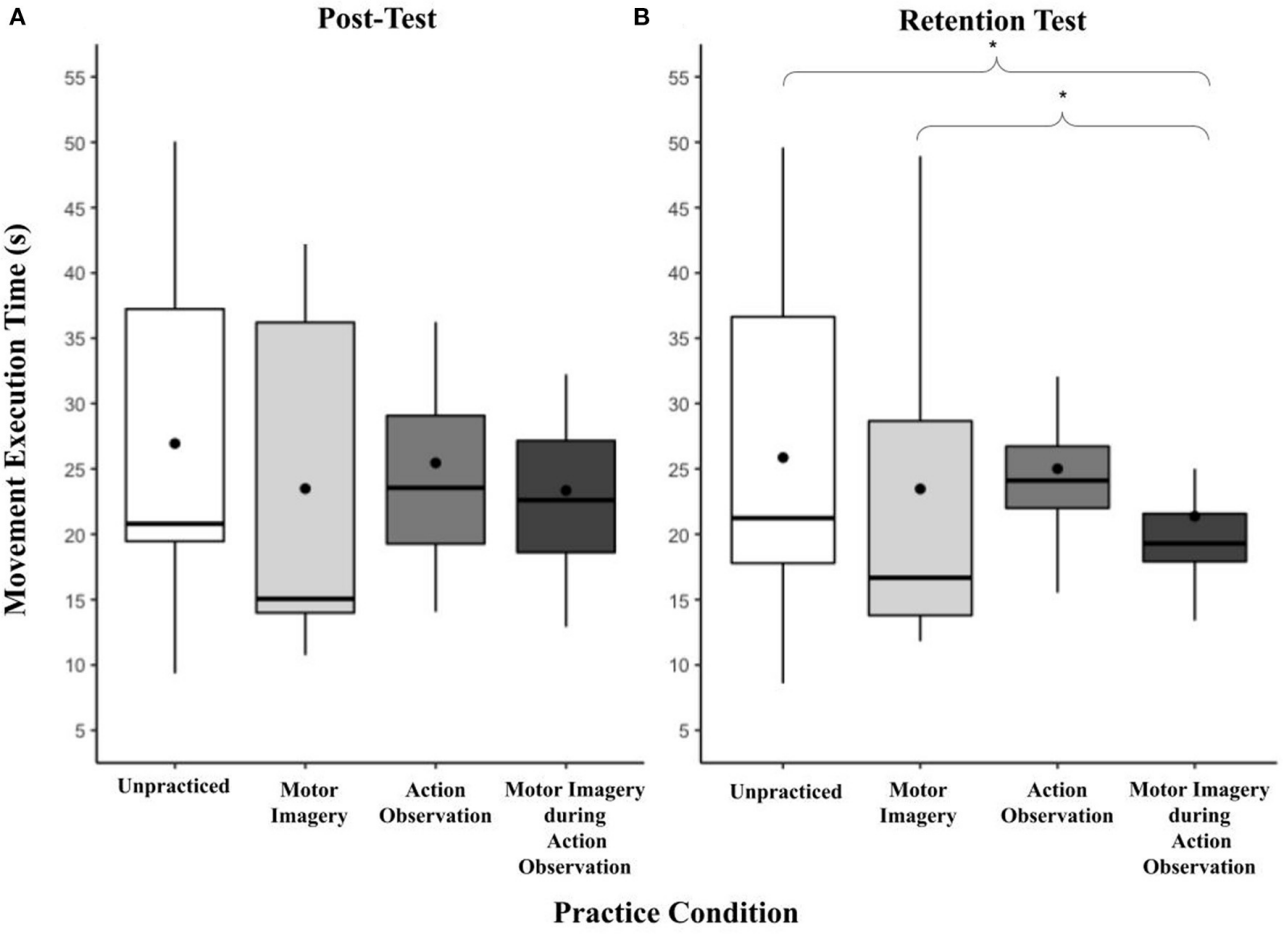
**(A, B)** Box and whisker plot displaying movement execution times at post and retention for practice condition. This figure shows the distribution of movement execution time data and skewness for all practice conditions at the post and retention test time points. These data are presented by displaying a six-number summary including: the minimum score (bottom of lowest line), first (lower) quartile (lower line), median (black horizontal line), third (upper) quartile (upper line), mean (black dot) and maximum score (top of upper line); ^*^p < 0.05.

There was a significant main effect of practice condition on mean movement execution times at the retention test, *F*_(3, 77)_ = 5.42, *p* < 0.01, $\eta_{p}^{2}$ = 0.17 (see Figure 4). AO + MI (*M* = 21.37, *SD* = 6.62) was significantly faster than MI [*M* = 23.47, *SD* = 12.89, *t*(87.2) = 3.08, *p* < 0.05] and the unpractised Control (*M* = 25.87, *SD* = 11.93, *t*(87.2) = 2.71, *p* < 0.05. While the mean movement execution times in the AO practice condition (*M* = 25.01, *SD* = 6.84) did not significantly differ from any other practice condition there was a close to significant finding in comparison to the MI practice condition; *t*(87.2) = 2.61, *p* = 0.051. No other practice conditions significantly differed from each other.

**Figure 5:**
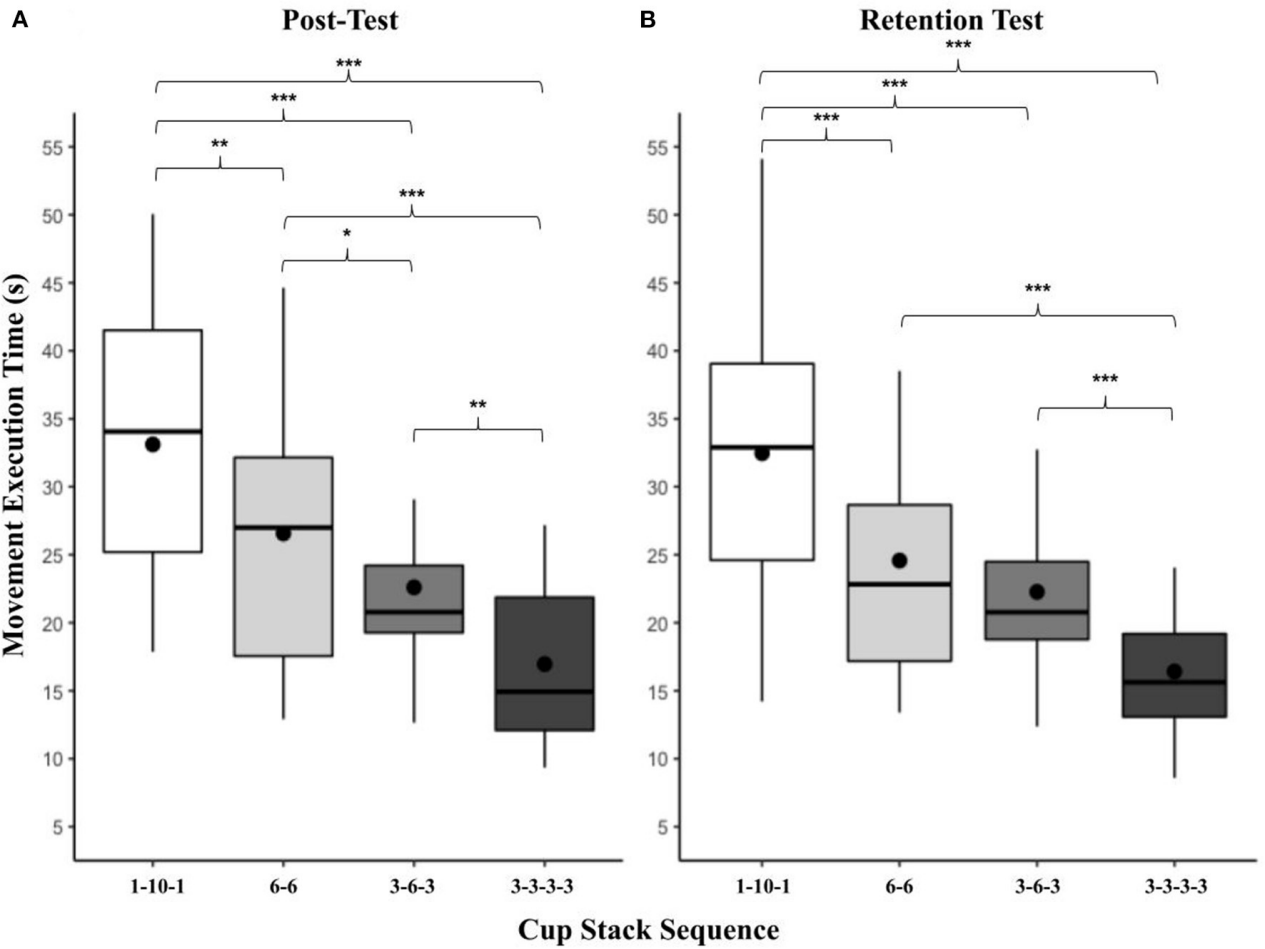
**(A, B)** Box and whisker plot displaying movement execution times at post and retention for task. This figure shows the distribution of movement execution time data and skewness for all cup-stack tasks at the post and retention test time points. These data are presented by displaying a six-number summary including: the minimum score (bottom of lowest line), first (lower) quartile (lower line), median (black horizontal line), third (upper) quartile (upper line), mean (black dot) and maximum score (top of upper line); ^*^p < 0.05; ^**^p < 0.01; ^***^p < 0.001.

A significant main effect of task was present at the retention test, *F*_(3, 77)_ = 69.84, *p* < 0.001, $\eta_{p}^{2}$ = 0.73 (Figure 5). The pattern replicated that of the two previous time points, in order from slowest to fastest: 1-10-1 (*M* = 32.47, *SD* = 10.10) > 6-6 (*M* = 24.57, *SD* = 9.52, *t(*87.2) = 5.57, *p* < 0.001) > 3-6-3 (*M* = 22.27, *SD* = 7.00) 3-3-3-3 (*M* = 16.41, *SD* = 5.52, *t(*87.2) = 4.99, *p* < 0.001). While 6-6 did not significantly differ from 3-6-3, all other task combinations significantly differed at *p* < 0.001. There was no significant effect of presentation order, *F*_(3, 77)_ = 1.79, *p* = 0.16, $\eta_{p}^{2}$ = 0.07.

Finally, there was a significant main effect of group, *F*_(3, 7)_ = 42.69, *p* < 0.001, $\eta_{p}^{2}$ = 0.95. Group 2 (*M* = 37.76, *SD* = 9.90) was significantly slower than Group 3 [*M* = 26.78, *SD* = 7.38, *t(*16.3) = 3.63, *p* < 0.05], group four (*M* = 20.69, *SD* = 6.97, *t(*16.3) = 5.98, *p* < 0.001 and Group 1 [*M* = 14.12, *SD* = 4.00, *t(*46) = 6.76, *p* < 0.001]. Group 3 was significantly slower than Group 1; *t(*16.3) = 4.18, *p* < 0.01. All other comparisons were not significant.

### Main effect of time

A mixed effect model, without interactions, was used to examine the main effect of time and the other variables (group, order, task, instruction, and time), *X*^2^ (14) = 136.01, *p* < 0.001. There was a significant main effect of time, *F*_(2, 278.67)_ = 22.29, *p* < 0.001, $\eta_{p}^{2}$ = 0.14. In comparison to the baseline (*M* = 43.45, *SD* = 36.59), mean times were significantly faster at the post-test [*M* = 24.81, *SD* = 10.29, *t(*291) = 5.44, *p* < 0.001] and the retention test (*M* = 23.93, *SD* = 9.96, *t*(291) = 5.86, *p* < 0.001). Post-test and retention test time points did not significantly differ from each other.

Interactions between time and the other variables were then added to the model, *X*^2^ (24) = 89.42, *p* < 0.001, ΔAIC −41.5. There was no significant interaction between time and practice condition, *F*_(6, 277.83)_ = 0.21, *p* = 0.97, $\eta_{p}^{2}$ = 0.00. There was a significant interaction between time and task, *F*_(6, 277.83)_ = 3.60, *p* < 0.01, $\eta_{p}^{2}$ = 0.07. While the differences between the mean execution times for the four tasks were larger at the baseline than at the post test and retention, the order remained the same at these two time points. There was a significant interaction between time and order, *F*_(6, 277.83)_ = 3.60, *p* < 0.01, $\eta_{p}^{2}$ = 0.07. While the order effect was significant at the baseline, this effect was not significant at both the post-test and retention. There was also a significant interaction between time and group, *F*_(6, 278.91)_ = 10.36, *p* < 0.001, $\eta_{p}^{2}$ = 0.18. This reflects that the effect of group was significant at the post and retention tests, but not significant at the baseline.

#### Interaction effects within the Graeco-Latin square design

An assumption of the Graeco-Latin square design is that of a null main effect for the Graeco factor (i.e., cup-stacking sequence). In the present study the cup-stacking sequences varied significantly in their associated movement execution times (reflecting differences in their inherent task complexity), which according to Kohli (107), does not permit a useful interpretation of the related interactions between the treatment factor (practice condition) and each of the other blocking factors (group and presentation order).

#### Additional outcome measures

##### Perceived impact of stroke

The results of the perceived impact of stroke (Stroke Impact Scale; SIS) are shown at baseline and retention test time points for individual participants in Table 3. Minimal detectable change and clinically important differences were observed, prior to and after the intervention, in all 8 domains and in total stroke recovery.

**Table 3:** Minimal detectable change and clinically important difference of the stroke impact scale in stroke patients.

**Participant**	**Strength**	**Memory**	**Emotions**	**Communication**	**ADL/ IADL**	**Mobility**	**Hand function**	**Participation**	**Stroke recovery**	**SIS score (baseline)**	**SIS score (retention)**
1	15^*^	3	24^*^	0	12^*^	13^*^	0	10	25^*^	339	441
2	10^*^	21^*^	9	0	0	5^*^	10	0	0	636	691
3	0	5	4	2	0	0	0	4	5	332	342
4	10^*^	17^*^	15^*^	14	20^**^	2	12	28^*^	20^*^	277	415
5	−5	−11	12	−9	6^*^	11^*^	0	−4	0	458	458
6	5	12	7	15^*^	−2	9^*^	20^*^	−2	15^*^	302	381
7	5	3	18^*^	0	−4	6^*^	0	0	0	621	649
8	0	0	5	3	12^*^	0	4	18^*^	0	637	679
9	5	−2	0	0	−14^*^	13^*^	0	−35^*^	20^*^	396	383
M (SD)	5.00 (6.12)	5.33 (9.91)	10.44 (7.57)	2.78 (7.46)	3.33 (10.25)	6.56 (5.22)	5.11 (7.29)	2.11 (17.42)	9.44 (10.44)	444.22 (149.82)	493.22 (139.46)

Individual change scores are presented in each domain. Total stroke recovery and total Stroke Impact Scale (SIS) score at Baseline and Retention time points. ^*^Clinically Important Differences (CIDs) and ^**^Minimal Detectable Changes (MDCs) are defined as the smallest change in an outcome measure that is perceived as beneficial to patients (109). ADL, activities of daily living; IADL, instrumental activities of daily living.

##### Motor imagery ability

Participants had significantly stronger imagery ability when they performed MI using their non-paretic limb at the baseline in comparison to their paretic limb (5.44 vs. 4.36; *F*_(1, 45)_ = 14.98; *p* < 0.001; $\eta_{p}^{2}$ = 0.25). This difference was maintained at the retention time point (5.41 vs. 4.45; *F*_(1, 45)_ = 26.81; *p* < 0.001; $\eta_{p}^{2}$ = 0.37). When these data were collapsed across limbs, there was no significant difference between the three MIQ-3 sub-scales at the baseline, and no significant improvement in ease of MI generation overall between the baseline and the retention test. However, at the retention test time point a significant difference was observed between the MIQ-3 subscales, *F*(2, 45) = 6.46; *p* < 0.001; $\eta_{p}^{2}$ = 0.22. The subscales that presented the weakest to strongest ease of imagery generation at retention are: kinaesthetic (*M* = 4.61, *SD* = 1.53) < external visual (*M* = 4.96, *SD* = 1.43) < internal visual imagery (*M* = 5.42, *SD* = 1.14). Internal visual imagery ability was significantly greater than kinaesthetic imagery at the retention time point, *t*(48.2) = 3.46, *p* < 0.01. Participants' imagery ability did not change from the baseline to the retention test time points, and the main effect of group was not significant in each analysis.

##### Upper extremity performance

The two-factorial ANOVA revealed a significant main effect of limb, *F*_(1, 105)_ = 45.23, *p* < 0.001, $\eta_{p}^{2}$= 0.30. Upper extremity performance was significantly better overall in the non-paretic compared to the paretic limb (*M* = 14.25, *SD* = 3.93 vs. *M* = 7.66, *SD* = 7.67). Neither the main effect of time, nor the two-way interaction were significant (see Table 4).

**Table 4:** Action research arm test (ARAT) scores at pre and retention test time points.

**Pre-Test**											
		**Grasp**		**Grip**		**Pinch**		**Gross movement**			
**Participant**	**Paretic side**	**Left**	**Right**	**Left**	**Right**	**Left**	**Right**	**Left**	**Right**	**Total left**	**Total right**
1	L	18	18	12	12	18	18	9	9	57	57
2	L	0	18	0	12	0	18	0	9	0	57
3	L	0	18	0	12	0	18	0	9	0	57
4	R	18	15	12	12	18	15	9	0	57	42
5	R	18	18	12	12	18	18	9	9	57	57
6	R	18	18	12	12	18	18	9	9	57	57
7	L	0	18	0	12	0	18	0	9	0	57
M (SD)		10.29 (9.62)	17.57 (1.13)	6.86 (6.41)	12.00 (0.00)	10.29 (9.62)	17.57 (1.13)	5.14 (4.81)	7.71 (3.40)	32.57 (30.47)	54.86 (5.67)
**Retention Test**											
		**Grasp**		**Grip**		**Pinch**		**Gross movement**			
**Participant**	**Paretic side**	**Left**	**Right**	**Left**	**Right**	**Left**	**Right**	**Left**	**Right**	**Total left**	**Total right**
1	L	18	18	12	12	18	18	9	9	57	57
2	L	0	18	0	12	0	18	0	9	0	57
3	L	0	18	0	12	0	18	0	9	0	57
4	R	18	15	12	12	18	18	9	0	57	45
5	R	18	18	12	12	18	18	9	9	57	57
6	R	18	18	12	12	18	18	9	9	57	57
7	L	0	18	0	12	0	18	0	9	0	57
M (SD)		10.29 (9.62)	17.57 (1.13)	6.86 (6.41)	12.00 (0.00)	10.29 (9.62)	18.00 (0.00)	5.14 (4.81)	7.71 (3.40)	32.57 (30.47)	55.29 (4.54)

The 19 items comprising the ARAT are scored using a 4-point ordinal scale, as follows: 0 = no movement, 1= movement task is partially performed, 2 = movement task is completed but takes abnormally long, 3 = movement is performed normally. Scores of < 10 points are considered poor, between 10 and 56 points–moderate and 57 points or above correlate with good recovery. Simpson and Eng (110) identified the minimal detectable change (MDC–at 95% confidence level) to be a change in score of 3.5. And van der Lee et al. (111) identified the minimally clinically important difference (MCID) in a defined chronic stroke population to be 10% of the total range of the measure (i.e., 5.7 points) and 12–17 points for a defined acute stroke population. None of the participants in the present study showed improvement in either limb that would meet the threshold defined as a MCD or a MCID.

##### Self-reported imagery use over time

Descriptive analyses show that ease of imagery generation was higher overall for AO + MI than for MI in both visual imagery (5.6 vs. 4.8, see Table 5) and kinaesthetic imagery (5.2 vs. 4.1) subscales. When averaged over both the visual and kinaesthetic items for both the AO + MI and MI practice conditions the ease of imagery generation improved slightly from Day 1 to Day 3 (5.4 vs. 4.5). Participants who reported spontaneous or unintentional MI in the AO practice condition also reported a reduction in frequency of imagery use from 57.14% of trials with spontaneous MI during AO on Week 2, to 14.29% on Week 6. In this subset of the data, imagery perspective also shifted from a 25% preference for an internal perspective on Week 2 to a 100% preference for an internal perspective on Week 6.

**Table 5:** Self-reported imagery use during training phase.

		**Motor imagery (MI)**			**Action observation** + **motor imagery (AO** + **MI)**			**Action observation (AO)**			
**Time Point**	**M SD**	**VI**	**KMI**	**Perspective used**	**VI**	**KMI**	**Perspective used**	**Confidence no imagery in AO**	**VI**	**KMI**	**Perspective used**
Week 2	M (SD)	4.00 (0.82)	3.43 (1.90)	85.71% internal	5.14 (0.90)	4.43 (1.72)	100% internal	57.14% unconfident	3.75 (1.71)	3.00 (1.15)	25% Internal
Week 4	M (SD)	5.00 (1.53)	4.00 (1.53)	85.71% internal	5.71 (0.95)	5.86 (1.07)	85.71% internal	42.86% unconfident	5.67 (0.00)	4.67 (0.00)	66.66% internal
Week 6	M (SD)	5.43 (0.98)	5.00 (1.53)	100% internal	5.86 (0.70)	5.29 (1.60)	100% internal	14.29% unconfident	6.00 (0.00)	5.00 (0.00)	100% internal

At three time points during the training phase (Week 2, 4, and 6), participants used the adapted MIQ-3 to rate their ease of generating visual and kinaesthetic MI, while also reporting their confidence in using either an internal or external perspective while observing the action on screen. Participants also rated their confidence in using AO without any confounding MI. Self-reported metrics were taken for MI, AO + MI and AO practice conditions. VI, Visual Imagery; KMI, Kinaesthetic Motor Imagery.

##### Social validation

Thematic analyses of the qualitative data generated three distinct themes, as described below.

###### Perceived impact

All participants who were available for the qualitative data collection after the study was completed (*n* = 9, 100%) reported that AO + MI was the most impactful and effective practice condition:

“*It was more believable and easier to associate with the video you could get your head around it, it was more realistic*,” Participant 8.

“*I was imagining how it would feel if my hands were doing it, so I was imagining the muscle in my bad arm lifting up, and the same in my other arm,”* Participant 4.

“*I could even imagine the noises with it*,” Participant 1.

“*It gave me a plan, and I could see what was expected and I had a good idea from the start, which for me I find most difficult—making a start*,” Participant 9.

The AO + MI condition was also the only condition that participants reported evoking or triggering any mental or physiological responses:

“*I could feel slight twitches, at the beginning I couldn't actually do that, but toward the end I could. I [also] noticed my finger, because I was imagining moving it, it was twitching*,” Participant 5.

###### Perceived difficulty

Most participants (56%) reported that AO was the most difficult:

“*There was a distraction. It was difficult to dissociate [the imagery] and only look for the dots*. *I was concentrating and thinking for it*,” Participant 5.

33% of participants believed the MI condition was the most difficult:

“*It felt too passive, I didn't feel involved with it*,” Participant 3.

“*When I was doing it, I was trying to match the speed and I was a little bit off keeping time, it was difficult*,” Participant 7.

One participant (11%) reported that AO + MI was the most difficult condition to undertake:

“*In the early stages getting your head around [AO* + *MI] was quite difficult but the more you got used to it, the easier it got. It felt as though it [AO* + *MI] used more brain cells, you had to think harder about it rather than just watching it*,” Participant 4.

###### Personal reflections on AO + MI therapy

Participants were invited to provide their general reflections on the activities undertaken during this study. These were largely positive in nature, with critical insight provided into the perceived usefulness and impact of this rehabilitation method in daily living:

“*I would [recommend it] it makes your brain tired, so I think something is working hard in your brain to fulfill that. What I was pleased about was that I have seemed to improve, I think something has gone in and stayed there*,” Participant 4.

“*It was tiring and frustrating at times, but I thoroughly enjoyed it, and it has made me realize that I am not as useless as I sometimes think I am. Physically I am doing all I can, but mentally is the hard one. Anything I can do to improve memory or planning structure is a plus … it can only be a plus*,” Participant 3.

“*I used to enjoy cooking before the stroke, but I haven't had the confidence to do it since the stroke. I think that now after this and I had a recipe and instruction I would have more confidence to follow it*,” Participant 1.

“*It's not about doing the motor activity of the task, but the state of mind and getting your head around the task—it's mind over matter*,” Participant 7.

“*I realize it was important to teach my brain how to do these things and it has worked, I believe*,” Participant 5.

## Discussion

This within-participant study was the first to investigate the extent to which a novel complex cup-stacking task can be learned in a stroke survivor population through different forms of mental practice (i.e., AO + MI, AO, and MI). We predicted that the combined AO + MI practice condition would reduce movement execution times for the cup-stacking task to a greater extent than both the AO and MI conditions and an unpractised control condition, at both the post-test and the retention test time points. In partial fulfillment of this prediction, a significant main effect of practice condition was found at the two-week retention test, while this was not found at the post-test. This specifically identified that AO + MI practice is the preferable combination for reducing movement execution times, compared to both the MI and control conditions in the absence of physical practice of this task. The results of this experiment therefore support the proposal that novel complex actions can be learned and retained, in a chronic stroke survivor population.

### The effects of practice condition on neurorehabilitation

At the baseline, post-test, and retention tests, neither the AO nor the MI practice condition yielded significantly faster cup-stacking times when compared with the unpractised control. Next, we offer interpretations of these two findings, before addressing the significant advantage for AO + MI training at the retention test.

#### Action observation effects

While undertaking the AO practice condition, participants were instructed to watch the on-screen cup-stacking sequence in a way that encouraged passive attention to the movement kinematics, rather than intentional imitation of the task. To control for the potential, confound of spontaneous or unintended MI during the pure AO condition, and to control for fluctuations in each participant's motivation and attention to the task across trials, participants were asked to attend to the occasional appearance of a colored dot. Notably, our results did not replicate Hebert's (93) significant finding for AO, which showed cup-stacking times reduced when healthy adult participants were instructed to either engage in physical practice prior to observation or observe the action before intentionally imitating the action. Instead, we replicate the finding for the pure AO condition reported in Binks et al. (77) that there was no significant reduction in movement execution times at either the post-test or the retention test. This presumably contrasts with Hebert's (93) finding because the AO instructions used in their study evoked a fundamentally different motor process, potentially due to spontaneous MI during AO. The impact of this potential confound was reduced in the present study.

The proposed benefit of AO, in regard to motor skill learning, is to enhance the structure of mental representations by specifying the sequencing and timing of basic action concepts (61). Research from Rüther et al. (112), for example, found that the action observation network, which comprises sensorimotor brain regions, was engaged when participants observed a novel object construction task from a visual picture matching cue or a partner who sat opposite. Crucially, while AO has been found to evoke activity in the areas of the brain that partially overlap with those responsible for movement execution (113), a limitation of AO is that it provides a visual representation of an action, without necessarily involving a sense of agency in the observer, nor promoting a focus on one's own body schema and the related kinaesthetic sensations of the observed action (56).

#### Motor imagery effects

In the present study, we similarly identified that MI practice did not produce a significant improvement in mean movement execution times compared with the unpractised control condition, at both the post-test and retention test. This finding is in line with the results of Welage et al.'s (24) meta-analysis and replicates the findings of Binks et al.'s (77) study for pure MI. While completing the MI practice condition, participants in the present study were instructed to imagine performing cup-stacking in an internal 1st person visual perspective and were also asked to maintain an emphasis on “feeling” the sensations associated with the imagined action. A strength of our approach was that the MI condition presented a series of still-images portraying the cup-stack sequence. This communicated a visual instruction for imagining the novel action in a realistic way without involving observation of a dynamic action. This also ensured for temporal congruence in MI across the MI and AO + MI conditions. Crucially, this further meant that the information used to convey the different tasks across the AO, MI, and AO + MI conditions were equitable, reducing the impact of “information” as a potentially confounding variable across these three conditions.

While previous research in a healthy population has demonstrated that kinaesthetic MI can enhance corticospinal excitability, as assessed using TMS (43, 44, 114), a limitation of MI in a stroke survivor population is that if an individual is unskilled, inexperienced or has a damaged neuronal network pertaining to the proposed task, activation of the brain regions involved in MI will likely be more bilateral and diffuse than when the individual has experience in performing the physical task, and the associated behavioral gains are limited (115, 116). MI practice has also been theorized to lack the core component of sensory feedback, which is an essential ingredient for a stroke survivor's ability to update the functional motor plan based on an error detection and correction mechanism (117). To further investigate this, Welage et al.'s (24) meta-analysis reviewed the effect that MI interventions had on 245 participants, over five studies. MI alone did not yield a positive effect on relearning upper extremity function after stroke. Encouragingly, those authors suggested future research should investigate the effect of performing imagery while receiving concurrent AO and explore if this would induce a greater effect on the upper limb functional recovery. In partial support of this proposal, in the present study ease of internal visual imagery was significantly greater than kinaesthetic imagery at the retention test only, indicating a potential change over time. Future research should, however, determine if such changes in fact reflect the natural variance occurring in MI ability in a stroke survivor population, or a worthwhile change.

#### Action observation during motor imagery effects

In contrast to the null effects reported for both the AO and MI conditions, the present within-participants experiment demonstrates that AO + MI practice was effective for the acquisition of a novel and complex motor skill in the absence of physical practice. This result is in line with previous between-group studies demonstrating beneficial practice effects for AO + MI training in neurotypical populations compared to AO [e.g., (118)], or MI [e.g., (71, 72)], or compared to both AO and MI (74–76). Building upon the significant findings of Binks et al. (77), the present study is the first to fully counterbalance the research design to control for several common sources of extraneous variability, while analyzing motor learning *via* mental practice in a stroke survivor population.

Lugassy et al. (119) found that procedural complex motor learning is stabilized and enhanced only after post-acquisition consolidation processes. In their study, gains in performance were only accumulated after a period of more than 24 h following skill acquisition and not after a 12-hour interval, despite also including sleep. Likewise, in the present study, participants who executed the task at the immediate post-test may not have had a sufficient period of learning consolidation (i.e., the 2-week retention period). They may also have experienced some fatigue at the post-test resulting from the mental practice undertaken on that day, which might have impacted the post-test findings.

The present study provides a continuation of support for the work of Sun et al. (81), Choi et al. (82), and Robinson-Bert and Woods (83), which similarly showed AO + MI instructions can enhance upper-extremity neurorehabilitation in stroke survivors. We extend their work by demonstrating that, despite the lower training dosage used in the current study, beneficial AO + MI effects were obtained following a 2-week retention period. Specifically, the main effect of practice condition was found at the two-week retention test, but not at the post-test.

This finding also aligns with recent stroke research showing AO + MI practice can improve the following: vascular cognitive impairments (84), activation and functional connectivity of brain regions involved in swallowing (120), and classification of performance in a brain-computer interface (121).

The main strength and proposed novelty of our research design was that the AO + MI practice condition was paired with each of the four different cup-stacking tasks, with different presentation orders in a fully counterbalanced way across the four groups. In the *post-hoc* analyses, where results were averaged over the factors of group, task and order, AO + MI produced significantly faster cup-stacking sequences than MI and the unpractised control at the retention test. We propose the following explanations for the enhanced task performance in the combined AO + MI practice condition, compared with the applications of AO and MI independently.

It has been stated that relevant and experience-dependent *practice*, which encourages the brain to create and reorganize functionally appropriate neural connections, is the crux of neurorehabilitation. MI may be a sub-optimal neurorehabilitation tool for this experience due to the limitations inherent in the self-generation of action-related feedback, crucial for updating, maintaining, or creating an accurate motor plan *de novo*. This approach may also reinforce neural connections within the parameters of existing self-taught compensatory strategies–all of which have been found to interfere with the rehabilitation of the damaged brain (122). More positively, it was proposed by Therrien et al. (123, 124) that while observing an action it may be possible to adjust errors in one's own existing forward model of the action in real-time, encouraging the damaged brain to reorganize, reallocate and shape its connections to match the intended observed action. To build upon this, the proposed benefit of AO + MI, for a stroke survivor, is the continuous opportunity for refining and updating the visually-guided components of the mental simulation (55–57), while scaffolding their kinaesthetic-imagery-driven simulation to match. The AO + MI instruction would theoretically drive neural responses that stimulate functionally accurate growth selection and synaptic reorganization patterns, thus providing a unique way to *practice* and maintain an internal motor representation of the observed action. In this way, AO + MI training is established as an advantageous method for motor skill acquisition in the absence of physical practice. This concept is supported by our data, whereby participants reported that they found the ease of generating imagery increased during AO + MI training in comparison to MI training in both the visual imagery and kinaesthetic imagery subscales.

While we did not study neurophysiological activity in the present study, it is unlikely that visual representations, without the activation of motor related processes, would significantly impact subsequent physical movement times (125). Wright et al. (61) used TMS to investigate the extent to which corticospinal excitability can be modulated in healthy adults through different forms of mental practice (i.e., AO + MI, AO, and MI) during a basketball free throw. This experiment also found the independent use of AO or MI did not significantly differ when compared with the control condition. During AO + MI, however, corticospinal excitability was significantly greater than both the AO and a control condition. These results indicate that a pronounced neurophysiological response occurs when we are instructed to practice AO + MI rather than practicing either AO or MI without the other [see (62)]. AO + MI may therefore promote functional connectivity and plasticity within the brain in a unique way, facilitating motor execution as learning progresses [see (126)]. Moreover, it is possible that the benefits found for combined AO + MI training resulted from a process whereby an AO-triggered and a MI-generated representation were both maintained either in parallel or were merged to consolidate motor processes and facilitate the early phase of motor relearning (56). Future research could now explore this proposal using brain imaging techniques in stroke survivors.

In our study, we used a passive form of AO that is not directly comparable to the instructions used in conventional AO therapy (e.g., “please observe and then imitate the target action”). While substantial evidence supports the use of AO therapy for promoting upper-limb recovery in stroke rehabilitation [see (20–24)], this approach does not routinely instruct patients on how to engage in MI during AO. Inevitably, some patients might therefore spontaneously engage their own motor system in an effortful way during AO, either consciously or unconsciously (i.e., spontaneous MI without clinical guidance), while others may not. For those who do spontaneously engage in AO + MI during AO therapy, there is either little or no guidance on how to optimize this concurrent MI process. Indeed, this overlooked issue may even contribute to the heterogeneity both in the rate and extent of upper-limb recovery *via* AO therapy. Instead, our approach was to experimentally tease apart the effects of passive (or “pure”) AO from a highly structured form of AO + MI. While our results indicate that the best way forward in rehabilitation practice is to augment AO therapy, with specific guidance tailored to patients on how to engage MI during AO, future research is now required to test the feasibility and efficacy of this proposal.

This approach does not preclude instances where practitioners may wish to alternate between AO and then MI [i.e., asynchronous AO and MI, (56)]. While research has shown that this approach can be more effective than using synchronous AO + MI for motor learning in healthy adults [e.g., (74–76)], the reverse pattern of results was found in stroke rehabilitation (81). In the studies of asynchronous AO and MI, however, the instructions did not aim to prevent spontaneous MI during the AO segments. If this had occurred, the design would amount to a more intense schedule alternating between AO + MI and MI, rather than plainly alternating between AO and MI (56). Future research should therefore explore whether this more intense dose of motor simulation is advantageous for rehabilitation. Indeed, it may be that the heterogeneity in brain injuries caused by stroke (see Table 1) will to some extent determine the suitability of different mental practice techniques for promoting rehabilitation. It is therefore necessary for future research to establish a more detailed mechanistic understanding of the neurophysiological effects of mental practice before such tailored recommendations can be made.

### Accounting for extraneous variables in the research design

A major strength of the present design was the ability to account for extraneous factors which influence the design, while carrying through the design into the analysis of the results. The present study utilized a Graeco-Latin square design, which allowed systematic control over four sources of extraneous variability. This design permitted investigation into all four factors: rows (group), columns (order), Latin letters (practice condition) and Greek letters (task). A strength of this counterbalanced design is that the tasks appear only once with each practice condition, ensuring each factor is statistically orthogonal to all other factors (i.e., rows and columns), thereby further reducing experimental error.

In relation to the four cup-stacking tasks, the present experiment incorporated sequences approved by the World Sport Stacking Association (WSSA). The cups are specially designed to be aligned as a pyramid (i.e., the inside left lateral adjunct of each cup with that of the next), in a predetermined sequence as fast as possible. This is a complex and controlled motor sequencing task, where error is reliably reflected in mean execution times (93–105, 107, 108, 112–115, 117–127).

At baseline, post and retention test time points the direction of significant differences between tasks replicated the findings of Binks et al. (77). The mean times for each cup-stacking task (i.e., Greek letters within the design) were significantly different from one another, which identified increasing complexity across the four tasks in the following order: 1-10-1 > 6-6 > 3-6-3 > 3-3-3-3. As revealed by the significant time by task interaction, the magnitude of these differences was largest at baseline compared to the other two time points and remained the same between post and retention test time points. Each task was given to each group in a different (and randomly allocated) presentation order. The strength of this design feature is that it allows researchers to control for experimental error by minimizing potential confounds of learning through sequence (i.e., an order effect) while modulating task complexity. A limitation of this design for the present study, however, is that the interaction effects within the Graeco-Latin square design cannot be tested, as they are confounded with the main effects (107). That is, the practice condition effects are derived from averaging performance across four tasks that differ in complexity from each other.

All participants were randomly assigned to one of four groups before participating in the present study. Once they were allocated to a random permutation of the Graeco-Latin Square (see Table 2), each group faced four unique combinations of the practice condition and cup-stack task pairings. A significant interaction effect between group and time was observed across the post-test and the retention test time points only. We offer two explanations for the effect that random pairings had on the overall quickness of Group 1 and the slowness of Group 2. Previously, we noted that modulating task complexity is desirable in the present design and population, as it is possible to control for experimental error by minimizing potential confounds of learning through sequence (i.e., an order effect). However, a restriction is that the Graeco-Latin square design assumes a null effect of the Greek letters (in this case; cup-stacking task). All participants in Group 1 experienced the most difficult task (1-10-1) in the first order with the most optimal practice condition (AO + MI), therefore enhancing the learning in this group in all the subsequent randomized permutations of task and practice condition pairings. This effect was magnified by the final order of Group 1 which contained the easiest task (3-3-3-3) paired with the unpractised control. Conversely, Group 2 contained the combination of the most difficult tasks with the slowest practice conditions (unpractised + 6-6; MI + 1-10-1).

We also analyzed the impact of presentation order on time taken to execute cup-stacking sequences. At the baseline, Order 1 was found to be slower than Order 2, 3 and 4. Unsurprisingly, this order effect indicates that at baseline (when results are averaged over the levels of: group, practice condition and task) participants were slowest in the cup-stack that they physically executed first, compared with the cup-stacks that they subsequently executed. The absolute mean difference between orders was Order 1 > Order 2 (4.0 s), Order 2 > Order 3 (10.1 s), and Order 3 > Order 4 (1.2 s). These results most likely reflect an initial “fast” learning phase that is typically associated with execution of a novel action in the very early stages of skill acquisition. This is evidenced in a wide array of behavioral and neurophysiological studies that have investigated the role of fast and slow experience-driven changes for the acquisition of skilled motor performance in novices [see (128)]. The significant time by order interaction revealed that this pattern of results was not replicated at the post or retention test, suggesting that learning was consolidated during the motor simulation training phase and between post-test and retention test time points (119). The significant main effect of time further verifies this conclusion as there was no significant main effect of order at the retention test.

Given the significant impact that order had at baseline, future research into genuine mental practice effects should similarly attempt to randomly permute task and treatment factors (such as practice condition), while ensuring the treatment factor is statistically orthogonal to all other factors in their design, such as group allocation or presentation order. Further research could also isolate and explore possible interactions between AO + MI instructions and task complexity. For example, in the context of the widely-researched principles of instructional design theory (129). Specifically, this theory states that learning is optimized when it is organized hierarchically from simple instructions, early in learning, to more complex instructions, later in learning and when it provides a meaningful context in which subsequent ideas can be integrated. An interesting question is whether AO + MI instructions provide a more holistic and meaningful context for motor learning, in which subsequent stimuli can be integrated to advance learning regardless of task complexity.

The semi-structured social validation interview was used to check for compliance with the intended manipulations, while gauging participants' perceptions and experiences of the training phase. Here important and largely positive statements provide critical insights into the perceived usefulness and impact of the rehabilitation method. Unanimously, AO + MI was perceived as the most impactful and effective practice condition. When participants were invited to reflect about the experience, the AO + MI training was recommended, in their opinions, as a worthwhile intervention. These qualitative insights underscore the importance of gathering user feedback for the purpose of tailoring future iterations of the AO + MI protocol to better suit stroke survivor characteristics. A main goal of neurorehabilitation is to improve the quality of participants' daily living, and this can be improved when user groups can contribute to the feasibility of the protocol design.

There are three main design considerations that are noteworthy in the present study. Firstly, it is feasible that if participants had been exposed to a longer training period, better cup-stacking performances may have been achieved. For reference, Schuster et al.'s (130) systematic review outlines best practice for motor imagery interventions. Their review of 133 studies found the average imagery intervention to last 178 min. The present study required participants to practice for only 75 min over 5 weeks, it is therefore telling that significant results were still obtained over this relatively short training duration.

Secondly, we monitored and discovered a degree of spontaneous and unintended imagery use in the pure AO practice condition. At Week 2 of training, only 57.14% of participants reported that they were confident they did not use imagery during AO. This happened although all participants were clearly instructed not to do so. Crucially, this did not lead to significant improvements in movement execution in the AO condition, relative to the other practice conditions at either the post-test or retention test. Spontaneous MI use in AO was reduced to 42.86% on Week 4, and 14.29% on Week 6. We investigated all forms of unintended imagery using a questionnaire, which was adapted from the MIQ-3 (86). Of the participants who reported unintended imagery use, these participants were also more likely to use an external 3rd person visual perspective in the AO condition compared to in both the MI and AO + MI conditions (Week 2 = 25% internal; Week 4 = 66.66% internal; Week 6 = 100% internal). Clearly, any imagery use during the AO condition was undesirable in this experiment; however, we highlight the importance of monitoring its use and, as such, this is one of the first behavioral studies to account for unintended imagery use during a motor learning study.

Finally, we required all participants to incorporate small hand raises while watching the video on each trial in the training phase. While the conventional viewpoint of imagery is still widely predicated upon participants remaining still throughout imagery practice, the last decade has revealed encouraging research into dynamic forms of imagery, which involves small physical movements to indicate imagery performance (131). As such, this feature of our design aimed to control for attention and adherence to the task, while encouraging both spatial and temporal motor congruence with the desired movement. This approach is further supported by Guillot et al.'s (132) most recent review of imagery practice, which highlights the positive influence that a more dynamic form of imagery can have on motor performance, learning and recovery.

It is worth noting that administering an additional baseline test, for example, 4 weeks prior to the start of the experiment would have helped to establish the functional stability of the participants' stage of recovery prior to the intervention. Given that our sample was between 6- and 65-months post first stroke onset, however, we can assume that these participants would all be in a relatively stable recovery phase, that is, unaffected by potentially confounding factors such as spontaneous early recovery. Moreover, all participants were instructed to maintain their normal physical activity routines throughout the duration of the experiment.

The present study worked with a population who experienced a stroke >2 years before the experiment, therefore, the ability in their non-paretic limb was unchanged and their ability in the paretic limb was formed. Significant advances in ARAT ability were unlikely because there was no physical practice between the baseline and post-test and there was no focus on improving the specific motor tasks contained within the ARAT. In addition, no observed improvement in ARAT ability would control for any potential confound of physical improvement. Despite this, analysis of the stroke impact scale yields some positive self-reported results in all domains of everyday functioning across participants that cover a variety of different health dimensions which are important to stroke survivors: strength, memory, emotions, communication, activities of daily living and instrumental activities of daily living (ADL/IADL), mobility, hand function, participation, and total stroke recovery (see Table 3).

## Conclusion

The main finding of this experiment is that combined AO + MI practice of a complex and novel cup-stacking task resulted in significantly shorter movement execution times in stroke survivors at retention relative to MI and an unpractised control condition. Individual participants also reported clinically important changes in quality of life (Perceived impact of stroke; Stroke Impact Scale) and positive experiences of the AO + MI therapy (social validation). These results prompt opportunities and future considerations in the design and delivery of training methods and interventions in neurorehabilitation. Based on the results in the present study, we propose that when physical practice is not suitable, combined AO + MI therapy could be a useful adjunct for neurorehabilitation in chronic stroke survivors. Future research is now required to test the feasibility and efficacy of this approach in a larger trial.

## Data availability statement

The raw data supporting the conclusions of this article will be made available by the authors, without undue reservation.

## Ethics statement

The studies involving human participants were reviewed and approved by Research Ethics Committee School of Social Sciences and Law, Teesside University. The patients/participants provided their written informed consent to participate in this study.

## Author contributions

JB contributed to the conceptualization, software, investigation, formal analysis, visualization, resources, and writing of the original draft. JE contributed to the investigation, software, data curation, resources, and writing—review and editing. MS contributed to the investigation, resources, and writing—review and editing. CW, PS, and DE contributed to the conceptualization, formal analysis, data curation, writing—review and editing, and supervision. All authors contributed to the methodology.

## References

[1] Tsao CW Aday AW Almarzooq ZI Alonso A Beaton AZ Bittencourt MS. Heart disease and stroke statistics-2022 update: a report from the american heart association. Circulation. (2022) 45:E153–639. 10.1161/CIR.0000000000001052 35078371

[2] Barker WH Mullooly JP. Stroke in a defined elderly population, 1967-1985. A less lethal and disabling but no less common disease. Stroke. (1997) 28:284–90. 10.1161/01.STR.28.2.284 9040676

[3] Hendricks HT van Limbeek J Geurts AC Zwarts MJ. Motor recovery after stroke: a systematic review of the literature. Arch Phys Med Rehabil. (2002) 83:1629–37. 10.1053/apmr.2002.35473 12422337

[4] Kwakkel G Kollen B Lindeman E. Understanding the pattern of functional recovery after stroke: facts and theories. Restor Neurol Neurosci. (2004) 22:281–99. 15502272

[5] Kwakkel G van Wegen EEH Burridge JH Winstein CJ van Dokkum LEH Alt Murphy M. Standardized measurement of quality of upper limb movement after stroke: consensus-based core recommendations from the second stroke recovery and rehabilitation roundtable. Neurorehabil Neural Repair. (2019) 33:951–8. 10.1177/1545968319886477 31660781

[6] Lai S-M Studenski S Duncan PW Perera S. Persisting consequences of stroke measured by the stroke impact scale. Stroke. (2002) 33:1840–4. 10.1161/01.STR.0000019289.15440.F2 12105363

[7] Fuchs E Flügge G. Adult neuroplasticity: more than 40 years of research. Neural Plast. (2014) 2014:541870. 10.1155/2014/541870 24883212 PMC4026979

[8] Kelley MS Steward O. Injury-induced physiological events that may modulate gene expression in neurons and glia. Rev Neurosci. (1997) 8:147–77. 10.1515/REVNEURO.1997.8.3-4.147 9548230

[9] Crofts A Kelly ME Gibson CL. Imaging functional recovery following ischemic stroke: clinical and preclinical fMRI studies. J Neuroimag. (2020) 30:5–14. 10.1111/jon.12668 31608550 PMC7003729

[10] Rossini PM Forno GD Rossini PM. Neuronal post-stroke plasticity in the adult Consequences of sleep deprivation and/or changes of sleep-wake cycle View project Brain topography of sleep stages View project Neuronal post-stroke plasticity in the adult. In: Restorative Neurology and Neuroscience. (2004). IOS Press. Available online at: https://www.researchgate.net/publication/8215372 (accessed February 01, 2022).

[11] Kleim JA Jones TA. Principles of experience-dependent neural plasticity: implications for rehabilitation after brain damage. J Speech Lang Hear Res. (2008) 51:1. 10.1044/1092-4388(2008/018) 18230848

[12] Wang H Xu G Wang X Sun C Zhu B Fan M. The reorganization of resting-state brain networks associated with motor imagery training in chronic stroke patients. IEEE Trans Neural Syst Rehabilitation Eng. (2019) 27:2237–45. 10.1109/TNSRE.2019.2940980 31536007

[13] Davies L Delcourt C. Current approach to acute stroke management. Intern Med J. (2021) 51:481–7. 10.1111/imj.15273 33890368

[14] Allred RP Kim SY Jones TA. Use it and/or lose it-experience effects on brain remodeling across time after stroke. Front Hum Neurosci. (2014) 8:379. 10.3389/fnhum.2014.00379 25018715 PMC4072969

[15] Garrison KA Aziz-Zadeh L Wong SW Liew S-L Winstein CJ. Modulating the motor system by action observation after stroke. Stroke. (2013) 44:2247–53. 10.1161/STROKEAHA.113.001105 23743974 PMC3753677

[16] de Vries S Mulder T. Motor imagery and stroke rehabilitation: a critical discussion. J Rehabilitation Med. (2007) 39:5–13. 10.2340/16501977-0020 17225031

[17] Maier M Ballester BR Verschure PFMJ. Principles of neurorehabilitation after stroke based on motor learning and brain plasticity mechanisms. Front Syst Neurosci. (2019) 13:74. 10.3389/fnsys.2019.00074 31920570 PMC6928101

[18] Ray M Dewey D Kooistra L Welsh TN. The relationship between the motor system activation during action observation and adaptation in the motor system following repeated action observation. Hum Mov Sci. (2013) 32:400–11. 10.1016/j.humov.2012.02.003 23632202

[19] Yoxon E Welsh TN. Motor system activation during motor imagery is positively related to the magnitude of cortical plastic changes following motor imagery training. Behav Brain Res. (2020) 390:112685. 10.1016/j.bbr.2020.112685 32428633

[20] Rizzolatti G Fabbri-Destro M Nuara A Gatti R Avanzini P. The role of mirror mechanism in the recovery, maintenance, and acquisition of motor abilities. Neurosci Biobehav Rev. (2021) 127:404–23. 10.1016/j.neubiorev.2021.04.024 33910057

[21] Simonsmeier BA Andronie M Buecker S Frank C. The effects of imagery interventions in sports: a meta-analysis. Int Rev Sport Exerc Psychol. (2021) 14:186–207. 10.1080/1750984X.2020.178062734049014

[22] Ste-Marie DM Lelievre N Germain L. Revisiting the applied model for the use of observation: a review of articles spanning 2011–2018. Res Q Exerc Sport. (2020) 91:594–617. 10.1080/02701367.2019.1693489 32004119

[23] Ryan D Fullen B Rio E Segurado R Stokes D O'Sullivan C. Effect of action observation therapy in the rehabilitation of neurologic and musculoskeletal conditions: a systematic review. Arch Rehabil Res Clin Transl. (2021) 3:100106. 10.1016/j.arrct.2021.100106 33778479 PMC7984987

[24] Welage N Bissett M Fong KNN Fahey P Coxon K Liu KPY. Effectiveness of action observation and motor imagery on relearning upper extremity function after stroke: a systematic review and meta-analysis. J Clin Neurosci. (2022) 9:e5–e5. 10.34172/icnj.2022.05

[25] Neuman B Gray R. A direct comparison of the effects of imagery and action observation on hitting performance. Mov Sports Sci–Sci Mot. (2013) 79:11–21. 10.1051/sm/2012034

[26] Vogt S Thomaschke R. From visuo-motor interactions to imitation learning: Behavioural and brain imaging studies. J Sports Sci. (2007) 25:497–517). 10.1080/02640410600946779 17365538

[27] Rizzolatti G Sinigaglia C. The functional role of the parieto-frontal mirror circuit: interpretations and misinterpretations. Nat Rev Neurosci. (2010) 11:264–74. 10.1038/nrn2805 20216547

[28] Borges LR Fernandes AB Melo LP Guerra RO Campos TF. Action observation for upper limb rehabilitation after stroke. Cochrane Database Syst Rev. (2018) 10:CD011887. 10.1002/14651858.CD011887.pub2 30380586 PMC6517007

[29] Mancuso M Tondo S di Costantini E Damora A Sale P Abbruzzese L. Action observation therapy for upper limb recovery in patients with stroke: a randomized controlled pilot study. Brain Sci. (2021) 11:3. 10.3390/brainsci11030290 33652680 PMC7996947

[30] Sarasso E Gemma M Agosta F Filippi M Gatti R. Action observation training to improve motor function recovery: a systematic review. Arch Physiother. (2015) 5:14. 10.1186/s40945-015-0013-x 29340183 PMC5759925

[31] Kim K. Action observation for upper limb function after stroke: evidence-based review of randomized controlled trials. J Physical Therapy Sci. (2015) 27:3315–7. 10.1589/jpts.27.3315 26644700 PMC4668191

[32] Zhang B Kan L Dong A Zhang J Bai Z Xie Y. The effects of action observation training on improving upper limb motor functions in people with stroke: a systematic review and meta-analysis. PLoS One. (2019) 14:8. 10.1371/journal.pone.0221166 31469840 PMC6716645

[33] Ertelt D Small S Solodkin A Dettmers C McNamara A Binkofski F. Action observation has a positive impact on rehabilitation of motor deficits after stroke. Neuroimage. (2007) 36:T164–73. 10.1016/j.neuroimage.2007.03.043 17499164

[34] Franceschini M Ceravolo MG Agosti M Cavallini P Bonassi S Dall'Armi V. Clinical relevance of action observation in upper-limb stroke rehabilitation: a possible role in recovery of functional dexterity. A randomized clinical trial. Neurorehabil Neural Repair. (2012) 26:456–62. 10.1177/1545968311427406 22235059

[35] Peng T-H Zhu J-D Chen C-C Tai R-Y Lee C-Y Hsieh Y-W. Action observation therapy for improving arm function, walking ability, and daily activity performance after stroke: a systematic review and meta-analysis. Clin Rehabil. (2019) 33:1277–85. 10.1177/0269215519839108 30977387

[36] Zhang JJQ Fong KNK Welage N Liu KPY. The activation of the mirror neuron system during action observation and action execution with mirror visual feedback in stroke: a systematic review. Neural Plast. (2018) 18:2321045. 10.1155/2018/2321045 29853839 PMC5941778

[37] Liu KP Chan CC Lee TM Hui-Chan CW. Mental imagery for promoting relearning for people after stroke: a randomized controlled trial. Arch Phys Med Rehabil. (2004) 85:1403–8. 10.1016/j.apmr.2003.12.035 15375808

[38] Zimmermann-Schlatter A Schuster C Puhan MA Siekierka E Steurer J. Efficacy of motor imagery in post-stroke rehabilitation: a systematic review. J Neuroeng Rehabil. (2008) 5:8. 10.1186/1743-0003-5-8 18341687 PMC2279137

[39] Jackson PL Lafleur MF Malouin F Richards CL Doyon J. Functional cerebral reorganization following motor sequence learning through mental practice with motor imagery. Neuroimage. (2003) 20:1171–80. 10.1016/S1053-8119(03)00369-0 14568486

[40] Ladda AM Lebon F Lotze M. Using motor imagery practice for improving motor performance–a review. Brain Cogn. (2021) 150:105705. 10.1016/j.bandc.2021.105705 33652364

[41] Pascual-Leone A Nguyet D Cohen LG Brasil-Neto JP Cammarota A Hallett M. Modulation of muscle responses evoked by transcranial magnetic stimulation during the acquisition of new fine motor skills. J Neurophysiol. (1995) 74:1037–45. 10.1152/jn.1995.74.3.1037 7500130

[42] Wang X Wang H Xiong X Sun C Zhu B Xu Y. Motor imagery training after stroke increases slow-5 oscillations and functional connectivity in the ipsilesional inferior parietal lobule. Neurorehabil Neural Repair. (2020) 34:321–32. 10.1177/1545968319899919 32102610

[43] Grosprêtre S Lebon F Papaxanthis C Martin A. New evidence of corticospinal network modulation induced by motor imagery. J Neurophysiol. (2016) 115:1279–88. 10.1152/jn.00952.2015 26719089 PMC4808134

[44] Grosprêtre S Lebon F Papaxanthis C Martin A. Spinal plasticity with motor imagery practice. J Physiol. (2019) 597:921–34. 10.1113/JP276694 30417924 PMC6355716

[45] Sharma N Baron J-C Rowe JB. Motor imagery after stroke: relating outcome to motor network connectivity. Ann Neurol. (2009) 66:604–16. 10.1002/ana.21810 19938103 PMC3791355

[46] Cumming J Eaves DL. The nature, measurement, and development of imagery ability. Imagin Cogn Pers. (2018) 37:375–93. 10.1177/0276236617752439

[47] Eaves DL Emerson JR Binks JA Scott MW Kenny RPW. Imagery ability: the individual difference gradient and novel training methods (Commentary on Kraeutner et al. (111)). Eur J Neurosci. (2018) 47:10. 10.1111/ejn.13928 29729203

[48] Kilteni K Andersson BJ Houborg C Ehrsson HH. Motor imagery involves predicting the sensory consequences of the imagined movement. Nat Commun. (2018) 9:1617. 10.1038/s41467-018-03989-0 29691389 PMC5915435

[49] Barclay RE Stevenson TJ Poluha W Semenko B Schubert J. Mental practice for treating upper extremity deficits in individuals with hemiparesis after stroke. Cochrane Database Syst Rev. (2020) 5:CD005950. 10.1002/14651858.CD005950.pub5 32449959 PMC7387111

[50] Braun S Kleynen M van Heel T Kruithof N Wade D Beurskens A. The effects of mental practice in neurological rehabilitation; a systematic review and meta-analysis. Front Hum Neurosci. (2013) 7:390. 10.3389/fnhum.2013.00390 23935572 PMC3731552

[51] Monteiro KB Cardoso MDS Cabral VR da Santos dos AOB Silva PS da Castro JBP. Effects of motor imagery as a complementary resource on the rehabilitation of stroke patients: a meta-analysis of randomized trials. J Stroke Cerebrovascular Dis. (2021) 30:105876. 10.1016/j.jstrokecerebrovasdis.2021.105876 34049014

[52] Butler AJ Page SJ. Mental practice with motor imagery: evidence for motor recovery and cortical reorganization after stroke. Arch Phys Med Rehabil. (2006) 87:2–11. 10.1016/j.apmr.2006.08.326 17140874 PMC2561070

[53] Machado S Lattari E de Sa A Rocha N Yuan T-F Paes F. Is mental practice an effective adjunct therapeutic strategy for upper limb motor restoration after stroke? a systematic review and meta- analysis. CNS. (2015) 14:567–75. 10.2174/1871527314666150429112702 25921745

[54] Machado S Lattari E Paes F Rocha NBF Nardi AE Arias-Carrión O. Mental practice combined with motor rehabilitation to treat upper limb hemiparesis of post-stroke patients: clinical and experimental evidence. Clin Pract Epidemiology Ment. Health. (2016) 12:9–13. 10.2174/1745017901612010009 27346996 PMC4797678

[55] Eaves DL Riach M Holmes PS Wright DJ. Motor imagery during action observation: a brief review of evidence, theory and future research opportunities. Front Neurosci. (2016) 10:514. 10.3389/fnins.2016.00514 27917103 PMC5116576

[56] Eaves DL Hodges NJ Buckingham G Buccino G Vogt S. Enhancing motor imagery practice using synchronous action observation. Psychol Res. (2022). 10.1007/s00426-022-01768-7 36574019 PMC11315722

[57] Vogt S di Rienzo F Collet C Collins A Guillot A. Multiple roles of motor imagery during action observation. Front Hum Neurosci. (2013) 7:807. 10.3389/fnhum.2013.00807 24324428 PMC3839009

[58] Bruton AM Holmes PS Eaves DL Franklin ZC Wright DJ. Neurophysiological markers discriminate different forms of motor imagery during action observation. Cortex. (2020) 124:119–36. 10.1016/j.cortex.2019.10.016 31865262

[59] Macuga KL Frey SH. Neural representations involved in observed, imagined, and imitated actions are dissociable and hierarchically organized. Neuroimage. (2012) 59:2798–807. 10.1016/j.neuroimage.2011.09.083 22005592 PMC3254825

[60] Taube W Mouthon M Leukel C Hoogewoud H-M Annoni J-M Keller M. Brain activity during observation and motor imagery of different balance tasks: an fMRI study. Cortex. (2015) 64:102–14. 10.1016/j.cortex.2014.09.022 25461711

[61] Wright DJ Wood G Eaves DL Bruton AM Frank C Franklin ZC. Corticospinal excitability is facilitated by combined action observation and motor imagery of a basketball free throw. Psychol Sport Exerc. (2018) 39:114–21. 10.1016/j.psychsport.2018.08.006

[62] Chye S Valappil AC Wright DJ Frank C Shearer DA Tyler CJ. The effects of combined action observation and motor imagery on corticospinal excitability and movement outcomes: two meta-analyses. Neurosci Biobehav Rev. (2022) 143:104911. 10.1016/j.neubiorev.2022.104911 36349570

[63] O'Shea H. Mapping relational links between motor imagery, action observation, action-related language, action execution. Front Hum Neurosci. (2022) 16:984053. 10.3389/fnhum.2022.984053 36466617 PMC9716994

[64] Emerson JR Binks JA Scott MW W Kenny RP Eaves DL. Combined action observation and motor imagery therapy: a novel method for post-stroke motor rehabilitation. AIMS Neurosci. (2018) 5:4. 10.3934/Neuroscience.2018.4.236 32341964 PMC7179337

[65] Tani M Ono Y Matsubara M Ohmatsu S Yukawa Y Kohno M. Action observation facilitates motor cortical activity in patients with stroke and hemiplegia. Neurosci Res. (2018) 133:7–14. 10.1016/j.neures.2017.10.002 29031830

[66] Bek J Poliakoff E Marshall H Trueman S Gowen E. Enhancing voluntary imitation through attention and motor imagery. Exp Brain Res. (2016) 234:1819–28. 10.1007/s00221-016-4570-3 26892882 PMC4893065

[67] Eaves DL Haythornthwaite L Vogt S. Motor imagery during action observation modulates automatic imitation effects in rhythmical actions. Front Hum Neurosci. (2014) 8. 10.3389/fnhum.2014.00028 24600369 PMC3927126

[68] Scott MW Emerson JR Dixon J Tayler MA Eaves DL. Motor imagery during action observation enhances automatic imitation in children with and without developmental coordination disorder. J Exp Child Psychol. (2019) 183:242–60. 10.1016/j.jecp.2019.03.001 30921604

[69] Scott MW Emerson JR Dixon J Tayler MA Eaves DL. Motor imagery during action observation enhances imitation of everyday rhythmical actions in children with and without developmental coordination disorder. Hum Mov Sci. (2020) 71:102620. 10.1016/j.humov.2020.102620 32452437

[70] Marshall B Wright DJ Holmes PS Wood G. Combining action observation and motor imagery improves eye–hand coordination during novel visuomotor task performance. J Mot Behav. (2020) 52:333–41. 10.1080/00222895.2019.1626337 31185831

[71] Scott M Taylor S Chesterton P Vogt S Eaves DL. Motor imagery during action observation increases eccentric hamstring force: an acute non-physical intervention. Disabil Rehabil. (2018) 40:1443–51. 10.1080/09638288.2017.1300333 28322596

[72] Taube W Lorch M Zeiter S Keller M. Non-physical practice improves task performance in an unstable, perturbed environment: motor imagery and observational balance training. Front Hum Neurosci. (2014) 8:972. 10.3389/fnhum.2014.00972 25538598 PMC4255492

[73] Ruffieux J Mouthon A Keller M Mouthon M Annoni J-M Taube W. Balance training reduces brain activity during motor simulation of a challenging balance task in older adults: an fMRI study. Front Behav Neurosci. (2018) 12:10. 10.3389/fnbeh.2018.00010 29472847 PMC5810285

[74] Romano-Smith S Wood G Wright DJ Wakefield CJ. Simultaneous alternate action observation and motor imagery combinations improve aiming performance. Psychol Sport Exerc. (2018) 38:100–6. 10.1016/j.psychsport.2018.06.00331385636

[75] Romano-Smith S Wood G Coyles G Roberts JW Wakefield CJ. The effect of action observation and motor imagery combinations on upper limb kinematics and EMG during dart-throwing. Scand J Med Sci Sports. (2019) 29:1917–29. 10.1111/sms.13534 31385636

[76] Romano-Smith S Roberts JW Wood G Coyles G Wakefield CJ. Simultaneous alternate combinations of action-observation and motor imagery involve a common lower-level sensorimotor process. Psychol Sport Exerc. (2022) 63:102275. 10.1016/j.psychsport.2022.102275

[77] Binks JA Wilson CJ Van Schaik P Eaves DL. Enhancing motor learning without physical practice: the effects of combined action observation and motor imagery practice on cup-stacking speed. PsyArXiv. (2023). 10.31234/osf.io/w6x54 37665909

[78] Gregor S Saumur TM Crosby LD Powers J Patterson KK. Study paradigms and principles investigated in motor learning research after stroke: a scoping review. Arch Rehabil Res Clin Transl. (2021) 3:100111. 10.1016/j.arrct.2021.100111 34179749 PMC8211998

[79] Tretriluxana J Khacharoen S Hiengkaew V Prayoonwiwat N. Learning of the bimanual cup-stacking task in individuals with chronic stroke improved with dyad training protocol. J Med Assoc Thai. (2014) 97:S39–44. 25141525

[80] Tretriluxana J Taptong J Chaiyawat P. Dyad training protocol on learning of bimanual cup stacking in individuals with stroke: effects of observation duration. J Med Assoc Thai. (2015) 98:S106–12. 26387420

[81] Sun Y Wei W Luo Z Gan H Hu X. Improving motor imagery practice with synchronous action observation in stroke patients. Top Stroke Rehabil. (2016) 23:245–53. 10.1080/10749357.2016.1141472 27077982

[82] Choi J-B Yang S-W Ma S-R. The effect of action observation combined with motor imagery training on upper extremity function and corticospinal excitability in stroke patients: a randomized controlled trial. Int J Environ Res Public Health. (2022) 19:12048. 10.3390/ijerph191912048 36231353 PMC9566430

[83] Robinson-Bert K Woods AB. Effectiveness of synchronous action observation and mental practice on upper extremity motor recovery after stroke. Occupational Therapy in Health Care. (2022) 29:1–18. 10.1080/07380577.2022.2138675 36309807

[84] Liu W Li Z Xie Y He A Hao D Dong A. Effects of a combined motor imagery and action observation intervention on vascular cognitive impairment: a randomized pilot study. Am J Phys Med Rehabil. (2022) 101:358–66. 10.1097/PHM.0000000000001827 35302529

[85] Brooke P Bullock R. Validation of a 6-item cognitive impairment test with a view to primary care usage. Int J Geriatr Psychiatry. (1999) 14:936–40. 10556864

[86] Williams SE Cumming J Ntoumanis N Nordin-Bates SM Ramsey R Hall C. Further validation and development of the movement imagery questionnaire. J Sport Exer Psychol. (2012) 34:621–46. 10.1123/jsep.34.5.621 23027231

[87] Lyle RC. A performance test for assessment of upper limb function in physical rehabilitation treatment and research. Int J Rehabil Res. (1981) 4:483–92. 10.1097/00004356-198112000-00001 7333761

[88] Duncan PW Lai SM Bode RK Perera S DeRosa J. Stroke impact scale-16: a brief assessment of physical function. Neurology. (2003) 60:291–6. 10.1212/01.WNL.0000041493.65665.D6 12552047

[89] Montgomery DC. Design and Analysis of Experiments. (1984). New York, NY: Wiley.

[90] Cochran WG. Recent advances in mathematical statistics: recent work on the analysis of variance. J Royal Statistical Soc. (1938) 101:434. 10.2307/2980213

[91] Kirk R. Experimental Design: Procedures for the Behavioral Sciences. Thousand Oaks, CA: SAGE Publications, Inc. (2013). 10.4135/9781483384733

[92] Richardson JTE. The use of Latin-square designs in educational and psychological research. Edu Res Rev. (2018) 24:84–97. 10.1016/j.edurev.2018.03.003

[93] Hebert E. The effects of observing a learning model (or two) on motor skill acquisition. J Mot Learn Dev. (2018) 6:4–17. 10.1123/jmld.2016-0037

[94] Holmes PS Collins DJ. The PETTLEP approach to motor imagery: a functional equivalence model for sport psychologists. J Appl Sport Psychol. (2001) 13:60–83. 10.1080/10413200109339004

[95] Moreno-Verdú M Hamoline G Van Caenegem EE Waltzing BM Forest S Chembila-Valappil A. Guidelines for reporting action simulation studies (GRASS): proposals to improve reporting of research in motor imagery and action observation. PsyArXiv. (2022). 10.31234/osf.io/9vywr 37956956

[96] Scott MW Wright DJ Smith D Holmes PS. Twenty years of PETTLEP imagery: an update and new direction for simulation-based training. J Sport Exerc Psychol. (2022) 2:70–9. 10.1016/j.ajsep.2022.07.002

[97] Eaves DL Behmer LP Vogt S. EEG behavioural correlates of different forms of motor imagery during action observation in rhythmical actions. Brain Cogn. (2016) 106:90–103. 10.1016/j.bandc.2016.04.013 27266395

[98] Mulder M Nijland R. Stroke impact scale. J Physiother. (2016) 62:117. 10.1016/j.jphys.2016.02.002 26947003

[99] Jenkinson C Fitzpatrick R Crocker H Peters M. The stroke impact scale: validation in a UK setting and development of a SIS short form and SIS index. Stroke. (2013) 44:2532–5. 10.1161/STROKEAHA.113.001847 23868278

[100] Nijland R van Wegen E Verbunt J van Wijk R van Kordelaar J Kwakkel G. A comparison of two validated tests for upper limb function after stroke: the wolf motor function test and the action research arm test. Chin J Rehabil Med. (2010) 42:694–6. 10.2340/16501977-0560 20603702

[101] R Core Team. R: A Language Environment for Statistical Computing. Vienna, Austria: R Foundation for Statistical Computing. (2021). Available online at: https://www.R-project.org (accessed May 01, 2022).

[102] Schielzeth H Dingemanse NJ Nakagawa S Westneat DF Allegue H Teplitsky C. Robustness of linear mixed-effects models to violations of distributional assumptions. Methods Ecol Evol. (2020) 11:1141–52. 10.1111/2041-210X.13434

[103] Muth C Bales KL Hinde K Maninger N Mendoza SP Ferrer E. Alternative models for small samples in psychological research. Educ Psychol Meas. (2016) 76:64–87. 10.1177/0013164415580432 29795857 PMC5965574

[104] Fisher RA. The design of experiments (2nd ed.). Edinburgh, Scotland: Oliver & Boyd. (1937).

[105] Satterthwaite FE. An approximate distribution of estimates of variance components. Bulletin. (1946) 2:6. 10.2307/3002019 20287815

[106] Cohen J. Statistical Power Analysis for the Behavioral Sciences. New York, NY: Academic Press (1969). p. 101–5.

[107] Kohli R. Assessing interaction effects in latin square-type designs. Int J Res Marketing. (1988) 5:25–37. 10.1016/0167-8116(88)90014-6

[108] Braun V Clarke V. Using thematic analysis in psychology. Qual Res Psychol. (2006) 3:77–101. 10.1191/1478088706qp063oa

[109] Lin KC Fu T Wu CY Wang YH Liu J Hsieh CJ. Minimal detectable change and clinically important difference of the stroke impact scale in stroke patients. Neurorehabil Neural Repair. (2010) 24:486–92. 10.1177/1545968309356295 20053950

[110] Simpson LA Eng JJ. Functional recovery following stroke. Neurorehabil Neural Repair. (2013) 27:240–50. 10.1177/1545968312461719 23077144 PMC4486379

[111] van der Lee JH de Groot V Beckerman H Wagenaar RC Lankhorst GJ Bouter LM. The intra- and interrater reliability of the action research arm test: a practical test of upper extremity function in patients with stroke. Arch Phys Med Rehabil. (2001) 82:14–9. 10.1053/apmr.2001.18668 11239280

[112] Rüther NN Brown EC Klepp A Bellebaum C. Observed manipulation of novel tools leads to mu rhythm suppression over sensory-motor cortices. Behav Brain Res. (2014) 261:328–35. 10.1016/j.bbr.2013.12.033 24393742

[113] Caspers S Zilles K Laird AR Eickhoff SB. ALE meta-analysis of action observation and imitation in the human brain. Neuroimage. (2010) 50:1148–67. 10.1016/j.neuroimage.2009.12.112 20056149 PMC4981639

[114] Stinear CM Byblow WD Steyvers M Levin O Swinnen SP. Kinesthetic, but not visual, motor imagery modulates corticomotor excitability. ExpBrain Res. (2006) 168:157–64. 10.1007/s00221-005-0078-y 16078024

[115] Burianová H Marstaller L Sowman P Tesan G Rich AN Williams M. Multimodal functional imaging of motor imagery using a novel paradigm. Neuroimage. (2013) 71:50–58. 10.1016/j.neuroimage.2013.01.001 23319043

[116] Kraeutner SN McWhinney SR Solomon JP Dithurbide L Boe SG. Experience modulates motor imagery-based brain activity. Eur J Neurosci. (2018) 47:1221–9. 10.1111/ejn.13900 29512844

[117] Annett J. Motor imagery: perception or action? Neuropsychologia. (1995) 33:1395–417. 10.1016/0028-3932(95)00072-B 8584177

[118] Kawasaki T Tozawa R Aramaki H. Effectiveness of using an unskilled model in action observation combined with motor imagery training for early motor learning in elderly people: a preliminary study. Somatosensory Motor Res. (2018) 35:204–11. 10.1080/08990220.2018.1527760 30592442

[119] Lugassy D Herszage J Pilo R Brosh T Censor N. Consolidation of complex motor skill learning: evidence for a delayed offline process. Sleep. (2018) 41:9. 10.1093/sleep/zsy123 30215814

[120] Xiong H Chen J-J Gikaro JM Wang C-G Lin F. Activation patterns of functional brain network in response to action observation-induced and non-induced motor imagery of swallowing: a pilot study. Brain Sci. (2022) 12:1420. 10.3390/brainsci12101420 36291353 PMC9599111

[121] Rungsirisilp N Wongsawat Y. Applying combined action observation and motor imagery to enhance classification performance in a brain–computer interface system for stroke patients. IEEE Access. (2022) 10:73145–55. 10.1109/ACCESS.2022.3190798

[122] Jones TA Adkins DL. Motor system reorganization after stroke: stimulating and training toward perfection. Physiology (Bethesda, Md). (2015) 30:358–70. 10.1152/physiol.00014.2015 26328881 PMC4556825

[123] Therrien AS Bastian AJ. Cerebellar damage impairs internal predictions for sensory and motor function. Curr Opin Neurobiol. (2015) 33:127–33. 10.1016/j.conb.2015.03.013 25863011 PMC4786071

[124] Therrien AS Wolpert DM Bastian AJ. Effective reinforcement learning following cerebellar damage requires a balance between exploration and motor noise. Brain. (2016) 139:101–14. 10.1093/brain/awv329 26626368 PMC4949390

[125] Higuchi S Holle H Roberts N Eickhoff SB Vogt S. Imitation observational learning of hand actions: Prefrontal involvement and connectivity. Neuroimage. (2012) 59:1668–83. 10.1016/j.neuroimage.2011.09.021 21983182

[126] O'Shea H Moran A. Does motor simulation theory explain the cognitive mechanisms underlying motor imagery? a critical review. Front Hum Neurosci. (2017) 11:72. 10.3389/fnhum.2017.00072 28261079 PMC5313484

[127] Foerster RM Carbone E Koesling H Schneider WX. Saccadic eye movements in a high-speed bimanual stacking task: changes of attentional control during learning and automatization. J Vis. (2011) 11:9–9. 10.1167/11.7.9 21665985

[128] Karni A Meyer G Rey-Hipolito C Jezzard P Adams MM Turner R. The acquisition of skilled motor performance: fast and slow experience-driven changes in primary motor cortex. Proc Nat Acad Sci. (1998) 95:861–8. 10.1073/pnas.95.3.861 9448252 PMC33809

[129] Reigeluth CM Merrill MD Bunderson CV. The structure of subject matter content and its instructional design implications. Instructional Sci. (1978) 7:107–26. 10.1007/BF00121929

[130] Schuster C Hilfiker R Amft O Scheidhauer A Andrews B Butler J. Best practice for motor imagery: a systematic literature review on motor imagery training elements in five different disciplines. BMC Med. (2011) 9:75. 10.1186/1741-7015-9-75 21682867 PMC3141540

[131] Guillot A Moschberger K Collet C. Coupling movement with imagery as a new perspective for motor imagery practice. Behav Brain Funct. (2013) 9:8. 10.1186/1744-9081-9-8 23425312 PMC3599464

[132] Guillot A Rienzo F di Frank C Debarnot U MacIntyre TE. From simulation to motor execution: a review of the impact of dynamic motor imagery on performance. Int Rev Sport Exerc Psychol. (2021) 1–20. 10.1080/1750984X.2021.2007539

